# Molecular Biology of *Escherichia coli* Shiga Toxins’ Effects on Mammalian Cells

**DOI:** 10.3390/toxins12050345

**Published:** 2020-05-23

**Authors:** Christian Menge

**Affiliations:** Friedrich-Loeffler-Institut/Federal Research Institute for Animal Health, Institute of Molecular Pathogenesis, Naumburger Str. 96a, D-07743 Jena, Germany; christian.menge@fli.de; Tel.: +49-3641-804-2430

**Keywords:** Shiga toxin, verotoxin, STEC, EHEC, O157, receptor, cytotoxicity, apoptosis, modulation

## Abstract

Shiga toxins (Stxs), syn. Vero(cyto)toxins, are potent bacterial exotoxins and the principal virulence factor of enterohemorrhagic *Escherichia coli* (EHEC), a subset of Shiga toxin-producing *E. coli* (STEC). EHEC strains, e.g., strains of serovars O157:H7 and O104:H4, may cause individual cases as well as large outbreaks of life-threatening diseases in humans. Stxs primarily exert a ribotoxic activity in the eukaryotic target cells of the mammalian host resulting in rapid protein synthesis inhibition and cell death. Damage of endothelial cells in the kidneys and the central nervous system by Stxs is central in the pathogenesis of hemolytic uremic syndrome (HUS) in humans and edema disease in pigs. Probably even more important, the toxins also are capable of modulating a plethora of essential cellular functions, which eventually disturb intercellular communication. The review aims at providing a comprehensive overview of the current knowledge of the time course and the consecutive steps of Stx/cell interactions at the molecular level. Intervention measures deduced from an in-depth understanding of this molecular interplay may foster our basic understanding of cellular biology and microbial pathogenesis and pave the way to the creation of host-directed active compounds to mitigate the pathological conditions of STEC infections in the mammalian body.

## 1. Introduction

Enterohemorrhagic *Escherichia coli* (EHEC), a subset of Shiga toxin-producing *E. coli* (STEC), are food-borne pathogens that can evoke life-threatening diseases, such as hemorrhagic colitis (HC) and hemolytic-uremic syndrome (HUS), in humans [1]. STEC strains producing the Shiga toxin 2e variant cause edema disease (ED) in piglets [2]. The pathogenesis of STEC-associated diseases originates from colonization and multiplication of the pathogens at intestinal mucosal surfaces. STEC strains, including the highly virulent O104:H4 strain which caused the large outbreak of HUS and HC in Germany in 2011, are not invasive [3,4,5]. Despite the fact that viable bacteria were occasionally found at necropsy in mesenteric lymph nodes in natural hosts [6], STEC cannot be detected in extra-intestinal tissues in the course of systemic disease manifestations [7,8]. Shiga toxins (Stxs), potent bacterial exotoxins produced and released by STEC, represent the principal virulence factors implicated in pathogenesis [9].

For EHEC-associated human diseases, the following model is generally considered [9,10,11,12]: Many EHEC strains inherit the ability to settle on the enteric mucosa by inducing attaching and effacing (AE) lesions, leading to tight association of single bacteria or small size colonies to the intestinal epithelial cells. These alterations are primarily independent of the Stxs‘ effects [13] and encoded by the locus of enterocyte effacement (LEE) in the STEC chromosome [14,15]. While the LEE is a key and prominent molecular determinant in pathogenesis, neither all EHEC nor STEC contain the LEE, indicating that some strains deploy additional virulence and colonization factors [16]. Stxs are produced by the pathogens during colonization and replication [5,17] and become released as free proteins liberated from the periplasmic space of the Gram-negative cell wall [18] or enclosed in outer membrane vesicles released by the bacteria [19]. Even in the absence of canonical Stx receptors on intestinal epithelial cells, luminal Stx facilitates the damage of the intestinal barrier indirectly, i.e., via effects on the underlying lamina propria [20], or by direct means because Stx2, but not Stx1, damages crypt epithelial cells [21]. The histological appearance of the tissue damage, manifesting mainly in the cecum and colon, is dominated by focal, intimate adhesion of the bacteria to the epithelial cells at the villus tips. The microvilli of the brush border are thickened or fused to each other or effaced from the apical cell poles of enterocytes. Attachment sites are underlaid by massive intracellular aggregates of cytoskeletal components. The regular arrangement of cells is disturbed, and ulceration occurs [13]. The loss of mature, fully differentiated epithelial cells is partially compensated for by immature epithelial cells. Fibrin exudation and hemorrhage is present in the submucosa. Neutrophilic infiltration is frequently found in the altered intestinal wall [22,23,24,25]. Because of the damaged epithelial layer [26], the transmigration of granulocytes [27] and by active Gb_3_/CD77-receptor-independent transport processes [28,29,30], Stxs reach the subepithelial layers of the intestinal wall [28], inducing a thrombotic microangiopathy in capillaries and arterioles. Augmented adherence of the highly virulent O104:H4 strain to intestinal epithelium, lacking the LEE locus but possessing the pAA virulence plasmid and expressing the corresponding phenotype of aggregative adherence to intestinal epithelial cells, might also facilitate systemic absorption of Stxs [3]. Swelling of the endothelial cells, in synergy with a widening of the subendothelial space, results in constriction of the vessel lumen, frequently clogged by thrombi. Smooth muscle cells in the tunica media may also be affected by necrotic processes. The proximity of the vessels is characterized by edema or hemorrhage [25]. These alterations are believed to be causative to the hemorrhagic character of HC. Bound to erythrocytes [31], neutrophils [32], platelets [33], or within host blood cell-derived microvesicles [34] in the blood stream, Stxs circulate through the entire body presumably accompanied by endotoxemia [35]. Subsequently, organ damage outside the gastrointestinal tract develops. Endothelial cells of the kidneys and the central nervous system are directly targeted by the Stxs [25]. Induction of a microangiopathy in the capillaries of the respective organs [25] is followed by edema and hemorrhage of the affected organ and ischemic damage to the functional organ tissue, e.g., necrosis of the renal glomeruli and tubuli in case of HUS [25,36,37]. Besides direct effects of the Stxs on endothelial cells, comprehensively reviewed by Bauwens et al. [38], evidence exists that the toxins also directly act on neuronal cells [39] as well as innate [40,41] and adaptive immune cells [42]. While the clinical meaning of the former remains elusive [43], the presumptive importance of the latter effects recently became appreciated [9].

The epidemiological link between STEC infections and development of HUS was established in 1985 [44]. Nevertheless, therapeutic options to treat human patients suffering from STEC-associated diseases are still limited at present and, if available [45], not directly counteracting the detrimental effects of the Stxs. Options to protect exposed human individuals against the development of Stx-induced diseases are not available [46] despite the fact that vaccination of piglets against Stx2e proved to be an effective strategy against ED in affected farms [47]. Preventive measures in place to mitigate the human health threat are implemented for the food chain (following the farm-to-fork concept) but are mainly limited to later stages of the chain (post-harvest food safety). Currently, no measures are effectively targeting wildlife and livestock ruminants which are the most important STEC reservoir [48]. Interestingly, Stxs are increasingly utilized as biotechnological tools to study cellular processes [49] and to develop novel therapeutic strategies for cancer treatment [50,51], adding Stxs and derivatives thereof to the toolbox for host directed therapy.

The recent unprecedented HUS outbreak in Europe 2011, caused by an unusual hybrid strain of the serotype O104:H4, which lacked the LEE locus [3], stresses the fact that Stxs are the principal virulence factors and the only common denominator of STEC strains posing a threat to human health. Consequently, novel efforts to counteract STEC must be directed against the Stxs [52,53] and all bacterial strains harboring the respective genetic information as the primary target [14,54]. Such targeted attempts apparently cannot be circumvented by tackling other STEC factors, i.e., vaccinating ruminants with antigens implicated in STEC adhesion [55] and iron metabolism [56]. Irrespective of significant species and tissue differences in cell susceptibility and tissue distribution of receptors, a comprehensive knowledge of the molecular mechanisms of Stx–host interactions needs to be considered to mitigate human health risk.

## 2. Variants and Molecular Structure of Shiga Toxins

The family of Stxs was named after the cytotoxin of *Shigella dysenteriae* type 1, a cytotoxin known since the early nineteen-hundreds. Like some other bacterial toxins (Cholera toxin (CT) of *Vibrio cholerae*, heat-labile enterotoxin (LT) of *Escherichia coli*), Stxs belong to the heteromeric protein toxins, consisting of one active (StxA-; 32 kDa) and five receptor-binding (StxB-; 7.7 kDa) subunits [1]. In 1977, Konowalchuk et al. [57] described a cytotoxin of an *Escherichia coli* (*E. coli*) isolate, for which, according to its toxicity for Vero cells, the term Vero(cyto)toxin was coined. O’Brien and LaVeck confirmed its close relationship to the Shigella cytotoxin [58]. Since then, several cytotoxins produced by *E. coli* and resembling the Shigella toxin have been discovered and can be assigned to one of two groups. The first comprises the *Shigella dysenteriae* toxin and the prototypic Stx1 of *E. coli* (also referred to as Stx1a; see Scheutz et al. [59]), at protein sequence level differing by only one amino acid [60]. In this review, reference is made to a number of studies having been conducted before introduction of the novel nomenclature. It is almost impossible to assign the current nomenclature to the toxins used in the cited studies in retrospect. For this reason, the author refrained from using the novel designation rather refers to the prototypic toxins as “Stx1” and “Stx2” throughout. Further variants, Stx1c [61] and Stx1d [62], possess a 91%–95% homology to Stx1 at the nucleotide sequence level. The second group, being only approximately 56% homologous to Stx1 at nucleotide sequence level, comprises the prototypic Stx2 (also referred to as Stx2a) and its variants Stx2c [63], Stx2d [64], Stx2e [65], Stx2f [66], and Stx2g [67]. Antisera against variants are partially cross-protective within but not between toxin groups [68]. Stx2e, the causative agent of ED in piglets is most distantly related to the other Stx2 variants in terms of protein sequence, biological activity, and receptor usage [69,70]. Genes for Stx1, Stx2, and Stx2 variants are encoded within the STEC genome as part of the genome of lambdoid bacteriophages [71]. STEC strains are often capable of producing more than one toxin type because possession of several *stx*-converting phages is common. Phages foster horizontal transmission of *stx* genes between different *E. coli* strains as well as to *Citrobacter freundii* and *Enterobacter cloacae* strains [72].

The *stx* genes encode for two polypeptide chains forming the toxin subunits. Translation products each consist of a peptide chain for the mature subunit, preceded by an N-terminal signaling sequence of 22 (StxA) and 19-20 (StxB) amino acids, respectively [63,73,74]. Upon transfer into the periplasmic space of the *E. coli* cell, the signal sequences are cleaved off by the membrane bound signal peptidase I [74].

**Figure 1:**
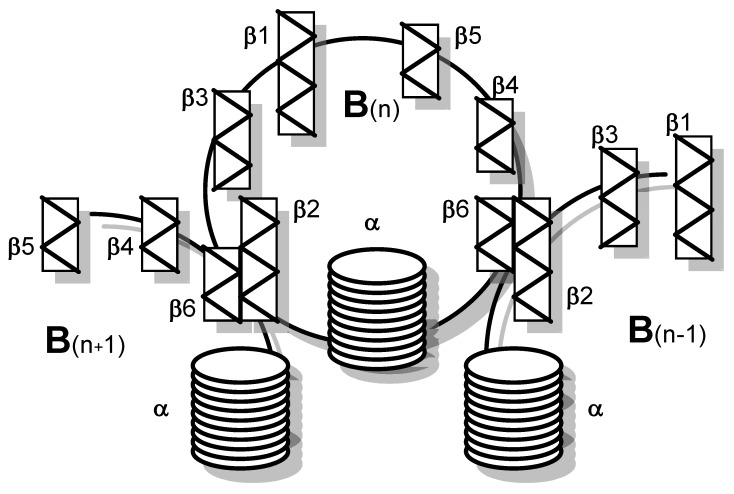
Simplified structural model of the Shiga toxin 1 B subunit as part of a pentamer (for details see text).

Five B subunits of Stx1 form a pentameric ring without establishing covalent bonds [75]. Each B subunit consists of 69 amino acids with an internal disulfide bond between cysteine residues at position 4 and 57 (Table 1) [76]. The Stx1 B subunit appears as a secondary structure with two three-stranded anti-parallel β-sheets, positioned to the outside of the pentamer [77]. Two three-stranded sheets of neighboring monomers build up a six-stranded β-sheet with the β2-sheet of one monomer interacting with the β6-sheet of the consecutive monomer via hydrogen bonds. Adjacent monomers form a pocket in which up to three potential receptor binding sites are located [78]. Each monomer also possesses an α-helix, directed to the center of the pentameric ring, which, together with the α-helices of the other four monomers, forms an 11 Å-wide pore (Figure 1) [77]. While the central α-helices undergo conformational changes at low pH, as it occurs upon endocytosis when the toxin reaches the endosomal compartment, the β-sheets forming the receptor binding sites on the B subunits’ outer surfaces are comparably stable [79].

**Figure 2:**
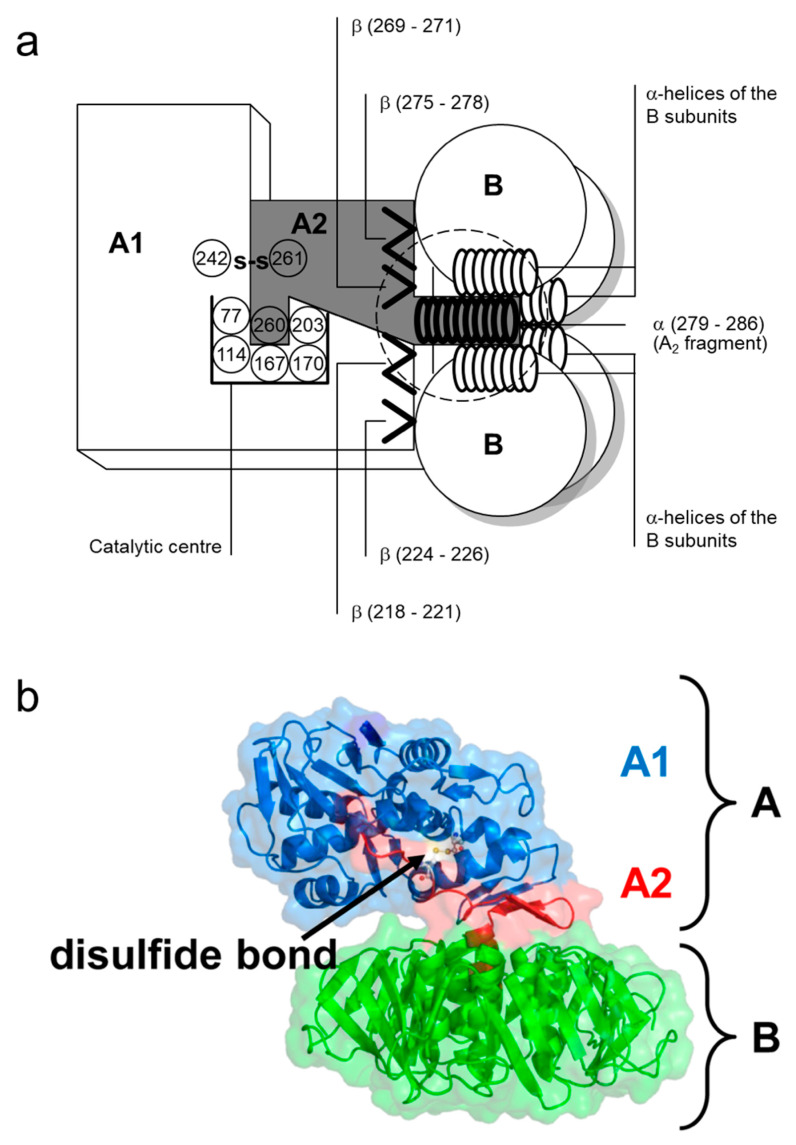
Structural model of Shiga toxin holotoxin. (**a**) Simplified scheme depicting the instrumental structural and functional elements of Stx1 and their approximate localization in the holotoxin (numbers in brackets refer to amino acid residues participating in formation of the structure), (**b**) Ribbon diagram of Stx2. The A_1_ fragment of the A subunit is colored in blue and the A_2_ fragment in red; the B5 pentamer is portrayed in green. The cysteine residues Cys242 and Cys261 form the disulfide bridge between the A_1_ and the A_2_ fragment. For a planar view on the surface of the B subunit pentamer the reader is referred to Figure 4a. Part b of Figure 2 reproduced from reference [92]. Elsevier, 2018.

The A subunit of Stx1 consists of 293 amino acids. Two cysteine residues in position 242 and 261 are connected by a disulfide bond [73]. In between, arginine residues at position 248 and 251 are part of a trypsin-sensitive cleavage site. Proteolytic cleavage separates the enzymatically active 27 kDa A_1_ fragment from the 6 kDa A_2_ fragment which is indispensable for the holotoxin structure [73,90]. Nine amino acids (residues 279–287) of the C-terminus of A_2_ form an α-helix, which is situated in an anti-parallel manner relative to the five α-helices of the B subunits surrounding it in the holotoxin [80,88,91]. The α-helix of A_2_ extends by 11 Å into the 20 Å-deep central pore of the B pentamer [80,88]. The other parts of the A subunit rest on the pentameric ring (Figure 2). Two β-sheets each of the A_1_ and the A_2_ fragment are non-covalently attached to the ring, building an asymmetric structure irrespective of the symmetric structure of the pentamer [80,88]. Establishment of this formation particularly relies on amino acids in position 277–278 and 288–289, which are located in vicinity to the α-helix of the A_2_ fragment. They are interlinked with charged and aromatic amino acids outside the pore on the planar surface of the B pentamer [91] such that the A subunit is interacting with three of the five B subunits in the holotoxin [80,88].

Toxins of the Stx2 group are comprised of an A subunit with 296–297 amino acids and B subunits with 68–70 amino acids [63,93,94]. Intramolecular disulfide bonds in the A subunit of Stx2 are formed between cysteine residues at position 241 and 260. The carboxy-terminus of the A subunit forms a short α-helix within the central pore of the B pentamer [80]. Amino acids essential for the holotoxin formation are highly conserved within the amino acid sequence of the Stx2 A subunit which is only 55% homologous to the A subunit of Stx1 [95], but the toxins exhibit further structural differences. As opposed to Stx1, in which the enzymatic cleft of the holotoxin is blocked by a methionine in position 260 of the A_2_ fragment [88], the cleft in Stx2 remains to be accessible for water molecules [80]. The tyrosine residue in position 77 further distinguishes the catalytic center of Stx2 from that of Stx1 [80]. The molecular mechanism of the enzymatic activity of all Stxs is nearly identical, even though the Stx2 A_1_ fragment has a higher affinity for and a faster association and dissociation with mammalian ribosomes than the Stx1 A_1_ fragment [96]. Irrespectively, differences in receptor binding sites of the particular B subunits, resulting in differences in the affinity of the toxins for the Stx receptor Gb_3_/CD77, are mainly held responsible for the differences in the relative in vitro and in vivo potency of Stx subtypes [97] and the type and degree of tissue alterations which are caused by Stx1- and Stx2-producing *E. coli* in humans, respectively [80].

Like other members of the AB_5_ family of bacterial exotoxins, Stxs are transiently localized in the periplasm before being secreted into the extracellular milieu, e.g., incorporated in bacterial outer membrane vesicles. If experimentally expressed in the absence of their cognate B subunits, the A subunits of Stxs and of heat-labile enterotoxin (LT) of enterotoxic *E. coli* (ETEC) were found to be degraded rapidly by periplasmic proteases, suggesting that the B subunit contributes to stability of the A subunit in the periplasm [98]. By contrast, a recent study suggests that the A and B subunits are not released as a holotoxin and that, unlike other AB_5_ toxin family members, Stxs are produced by STEC as unassembled A and B subunits. A preformed AB_5_ complex is not required for cellular toxicity or in vivo toxicity to mice, and toxin assembly is assumed to occur at the cell membrane [99]. Differences to other AB_5_ toxins in maintaining an intact AB_5_ conformation seems to be due to a small hydrophobic patch in the central pore of the B pentamer, which in other AB_5_ toxins is larger and plays a critical role in the engagement of the A subunit and the B pentamer [100].

## 3. Receptor Globotriaosylceramide (Gb_3_/CD77)

Globotriaosylceramide (Gb_3_) acts as the functional receptor for most Stxs. Because Gb_3_ is also present on immune cells, it became listed as the CD77 leukocyte antigen. Globotetraosylceramide (Gb_4_) was identified as the functional receptor for Stx2e. Of note, evidence exists that several binding sites varying in the affinity for Stxs do exist on host cells [83,101]. It was considered plausible that Gb_3_/CD77 and Gb_4_ are accompanied by non-functional receptors [102]. However, Gb_3_/CD77 does not exist as a chemically defined single molecular structure. It rather represents a group of glycosphingolipids, sharing a common carbohydrate group but significantly differing in the components constituting their lipid moiety. These lipid components impact the affinity of toxin receptor-binding as well as on the subsequent route the toxins are transported along into intracellular compartments. Consequently, toxin binding sites with varying affinity on cellular surfaces may reflect the presence of different Gb_3_/CD77 species, several of which, independent of their affinity, may all act as functional receptors [101].

### 3.1. Structure, Synthesis, and Regulation of Cell Surface Expression

**Figure 3:**
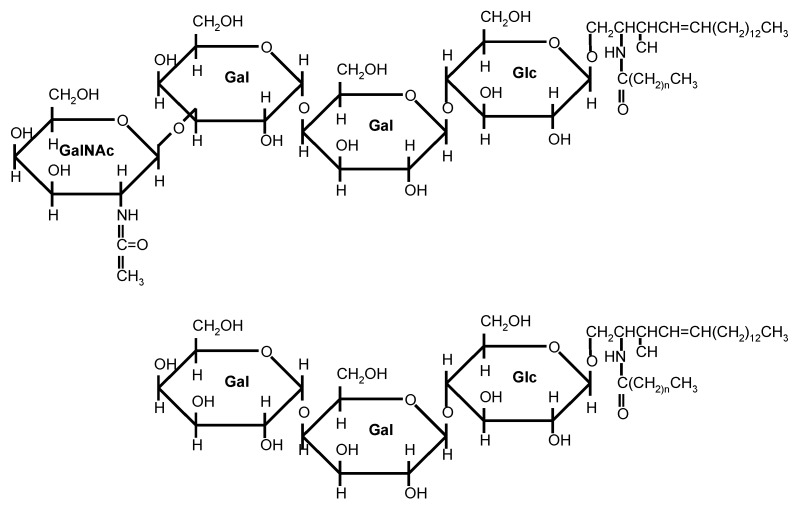
Molecular structure of globotetraosylceramide Gb_4_ (**top**) and globotriaosylceramide Gb_3_ (**bottom**).

Gb_3_/CD77 and Gb_4_ are neutral glycosphingolipids of the globo-series. The lipid parts of these molecules are composed of a ceramide, an amide-linkage between a sphingosine molecule, and a fatty acid. Neutral glycosphingolipids contain oligosaccharides that are attached to the terminal hydroxy group of the sphingosines in a β1-glycosidic manner [103]. By coupling glucose and galactose to ceramide, lactosylceramide (Gal β1-4Glcβ1-1Cer) is synthetized, the Stx receptor precursor [104]. Another galactose residue is added by the UDP-galactose:lactosylceramide α1-4-galactosyl transferase (Gb_3_-synthetase) to create Gb_3_/CD77 (Galα1-4Galβ1-4Glcβ1-1Cer) (Figure 3) [103,105,106]. Further addition of N-acetyl-galactosamine by the N-acetyl-galactosyl transferase results in formation of Gb_4_ (GalNAcβ1-3Galα1-4Galβ1-4Glcβ1-1Cer) [107].

For most toxin-sensitive cells, the presence of approximately 1 × 10^6^ to 1 × 10^7^ receptor molecules per cell has been estimated [102,108,109], however, expression on cells and in tissues depends on a number of factors. Active cellular division processes appear to be the principal prerequisite for maximum sensitivity of the target cells [110], as sensitivity varies with the phase of the cell cycle of the individual cell. Pudymaitis et al. [111] reported that Vero cell cultures exhibit highest sensitivity when in the transition from the G1 to the S phase, before sensitivity drops by one order of magnitude. In the G1 phase, maximum levels of Gb_3_/CD77 receptor synthesis and subsequent cell surface expression is accompanied by maximum binding of toxin molecules by the cells. Because the Gb_3_/CD77 content of the cultures remained rather constant during the cell cycle, an elevated surface exposition and receptor turnover was believed to facilitate toxin uptake [111]. By contrast, Majoul et al. [112] described that synthesis and surface expression of Gb_3_/CD77 reached highest levels between G2 and M phase. In turn, Stx1 irreversibly arrests HCT116 cells in the S phase within 24 h, presumably by activation of the S phase checkpoint prior to inducing apoptosis [113].

Glycosphingolipids are known to be detectable in cells yet not available for ligand binding at the cell surface. Their exposition not only depends on cellular functions, but their small head residues located close to the cell membrane can easily be masked by long-chained carbohydrates or proteins [111,114].

Surface expression of Gb_3_/CD77 also is a function of the degree of differentiation of cells and tissues. While THP-1 cells are Stx-sensitive in initial stages of differentiation, further stimulation in vitro by phorbol esters, interferon gamma (IFN-γ) or granulocyte-macrophage stimulating factor (GM-CSF) results in increasing resilience for Stx, accompanied by a reduced receptor expression [115]. The balance between synthesis of lactosylceramide to Gb_3_/CD77 by the Gb_3_-synthetase and degradation by the α-galactosidase regulates Gb_3_/CD77 expression on HeLa cells [104,114], but not on Daudi cells, implying the existence of different regulatory mechanisms [116].

Lipopolysaccharide (LPS) increases Stx receptor expression on human umbilical vein endothelial cells (HUVEC) by 10-fold and renders the cells more susceptible to Stx [117,118]. Similarly, tumor necrosis factor alpha (TNF-α), released from macrophages upon exposure to LPS or Stx, facilitates the effect of Stx on endothelial cells in an additive or synergistic manner. Pre-incubation of endothelial cells with TNF-α increases the number of Stx binding sites by up to 100-fold [118]. This increase in receptor expression results from an increased de novo synthesis following a protein kinase C- (PKC-)induced increased activity of the Gb_3_-synthetase [119] as well as an increased activity of the enzymes catalyzing the synthesis of precursor molecules [120]. Following LPS or TNF-α exposure, increases in receptor number become detectable after 6–8 hand remain for 48 h [119]. Interleukin 1 beta (IL-1β), also released from macrophages after LPS and Stx exposure, sensitizes endothelial cells to Stx with the same kinetics and to the same degree as TNF-α [121]. Via transcription factor NF-κB, IL-1β also induces the production of enzymes which degrade sphingomyelin molecules to ceramides, which in turn serve as substrate for Gb_3_/CD77 synthesis [121].

As opposed to HUVEC, human renal microvascular (HRMEC) and glomerular capillary endothelial cells (GCEC) become affected in vitro by Stx concentrations not sufficient to harm HUVEC [103,122]. As early as 5–6 h after addition of Stx1 to GCEC cultures, protein synthesis declines, resulting in cytopathic effects after 10 h [122]. Accordingly, renal endothelial cells possess a 50-fold higher Gb_3_/CD77 content as compared to HUVEC and at the same level as Vero cells [103]. The heterogeneity of endothelial cell responses to Stxs [103] also becomes apparent when considering the meaning of cytokines. While these immune system mediators neither influence receptor expression nor the cytotoxic effect of Stxs in renal endothelial cells in vitro [103,122], TNF-α and IL-1β drastically trigger receptor expression by microvascular endothelial cells from the human brain [123,124].

Under experimental conditions, Gb_3_/CD77 synthesis can be stimulated with butyric acid which induces cellular differentiation [109,125]. Butyric acid, a metabolic product of the anaerobic intestinal flora, may exhibit a similar effect in vivo on the intestinal epithelium.

### 3.2. Cellular and Tissue Distribution

Gb_3_/CD77 and Gb_4_ were detected in several cell lines, primary cultured cells and tissues of different host species with significant variations in tissue distribution between hosts (Table 2). Detection of Gb_3_/CD77 and Gb_4_, respectively, vastly correlates with the sensitivity of cells and tissues for the cytotoxic effect of Stxs, i.e., differences in clinical symptoms following infections with Stx2e and Stx1/2-producing *E. coli* correlate with receptor specificities of Stx2e versus other Stxs and the presence of Gb_4_ and Gb_3_/CD77, respectively, on cells and in tissues [22,69,83,126,127].

Stxs were initially recognized as cytolethal toxins but it became apparent later that the toxins cause a broad spectrum of cellular effects that vary with cell type. These merely modulating effects are now considered more important for the pathogenesis of STEC-mediated diseases than the cytolethal effect [11]. The kind of effect on a given cell type is determined by the localization of Gb_3_/CD77 in the cellular membrane [178]. In HeLa cells, highly susceptible to cytolethal effects of Stxs, Gb_3_/CD77 is embedded in detergent-insoluble, glycosphingolipid-rich microdomains within the cellular membrane, referred to as lipid rafts [178]. Gb_3_/CD77 located herein may trigger endocytosis and retrograde transport of Stxs to the biosynthetic/secretory vesicular transport path [178] as well as the activation of signaling cascades starting from the cell surface [179]. Composition of the lipid rafts may vary, as Gb_3_/CD77 is strictly colocalized with ganglioside GM_1_, a marker for lipid rafts, in polarized Caco-2 [180] and HeLa cells [178], but lipid rafts containing either GM_1_ or Gb_3_/CD77 are formed in different cell cycle phases in Vero cells [112].

Gb_3_/CD77 is located outside lipid rafts in human monocytes and macrophages [178], which are fairly resistant to the protein biosynthesis inhibition by Stx1 but respond with an altered cytokine transcription profile [157]. After disintegration of lipid rafts, Caco cells fail to internalize Stxs [180], whereas human monocytes still do so but primarily transport Stxs to late endosomes, where they become degraded before transferred to the cytosol [178].

### 3.3. Interactions with Shiga Toxins

#### 3.3.1. Binding Affinity and Kinetics

Binding of Stx holotoxin to Gb_3_/CD77 embedded in cellular membranes is characterized by a high association constant of 1 × 10^9^ to 1 × 10^10^ M^−1^ [108,130,181,182]. The binding constant for soluble trisaccharide to the soluble pentameric B subunit is weak, with a K(a) of 1 × 10^3^ M^−1^ for the B subunit monomer [183]. The holotoxins’ high avidity results from the multivalent property of Stxs for receptor binding by deploying up to 15 receptor binding sites. HeLa cells possess additional low affinity binding sites with high binding capacity, which does not correlate to the sensitivity of the cells for Stxs, though [102,181,184]. Because the affinity of Gb_3_/CD77 molecules is determined by the lipid part, the presence of Gb_3_/CD77 with different lipid moieties is considered causative for biphasic binding kinetics [101]. Other membrane lipids, referred to as auxiliary lipids, influence receptor affinity [185]. Stx1 and Stx2 seem to differ in the receptor binding mechanisms, which may contribute to different toxicities observed [126,186]. Stx binding is temperature-dependent with a maximum binding at 37 °C [102,187]. However, binding also rapidly occurs at 0 °C when internalization is prohibited. Fifty percent of maximum binding is reached after 5 min at 4 °C, 100% after 15 min [184]. The binding constant has been calculated as 1.5 × 10^6^ M^−1^s^−1^ [181]. Only 20% of cell-bound Stx1 is released again after 20 h at 0 °C [181]. Toxin binding also depends on pH with a broad plateau between pH 5–8 [184]; affinity for Gb_3_/CD77 only significantly decreases at pH-values below 3.5 [79].

#### 3.3.2. The Carbohydrate Moiety

Except for Stx2e, all Stxs specifically bind the terminal galabiose (Galα1-4Gal) of Gb_3_/CD77 in the membrane of eukaryotic cells [102,127,188,189]. Under artificial conditions, toxins also bind to isolated di- and trisaccharides [127], which are able to block cytolethal effects of Stx at equimolar concentrations [102,114,189]. Treatment of cells and tissues with α-galactosidase impairs toxin binding [69,188,189]. Under certain conditions, Gb_2_ (galabiosylceramide, Galα1-4Galβ1Cer) may serve as functional receptor [127,190]. Stx can bind to the P1 blood group antigen also harboring a terminal galabiose [102].

The Stx2e variant preferentially binds Gb_4_, with a galabiose located subterminal to n-acetyl-galactosamine [69]. Deacetylation of the carbohydrate does not influence Stx2e binding, indicating that the terminal β1-3 galactose structure is sufficient for binding [69]. The particular structure is also present in galactosyl-globotetrasylceramide (Gb_5_) and in the Forssman blood group antigen which also bind Stx2e [69,127].

#### 3.3.3. The Lipid Moiety

Stxs preferentially bind carbohydrate structures when the oligosaccharide is coupled to a lipid or protein [102]. In mammals, galabiose structures at the terminal, non-reduced end of saccharides have only been detected in glycolipids [114]. The lipid structure impacts on the presentation of the galabiose on the cell surface, i.e., Stx fails to bind to digalactosyldiglycerides [101,188]. The ceramide component of Stx receptors are composed of a sphingosine or dihydrosphingosine molecule to which a long-chained fatty acid is coupled via an amide bond [101]. Varying with the cellular source, Gb_3_/CD77 molecules may either harbor hydroxylated plus non-hydroxylated fatty acids [129,191] or non-hydroxylated fatty acids only [101].

The fatty acid length particularly influences the spatial orientation of the carbohydrate. Stxs preferentially recognize receptors harboring C12 to C24 fatty acids [101]. In thin layer chromatograms of glycolipids from human kidneys, Gb_3_/CD77 molecules present as two bands, consisting of a mixture of glycolipids with fatty acids of different lengths. One band is dominated by Gb_3_/CD77 molecules having incorporated a C24:1 fatty acid and binding Stx1 with low affinity but high capacity, the second band mainly consists of Gb_3_/CD77 molecules with a C16:1 fatty acid which bind Stx1 with high affinity and low capacity. Affinity and capacity of Stx1 binding to each of the bands is higher than binding to the respective semisynthetic receptor analog, indicating that a mixture of different receptor molecules with variable fatty acid content promotes Stx binding to physiological membranes. A combination of receptor variation and additional presence of auxiliary lipids may create an uneven surface, fostering binding of the StxB pentamer [101].

The affinity to Gb_3_/CD77 molecules with fatty acids of a given length seems to vary between different Stxs. Stx1 prefers Gb_3_/CD77 molecules with C20:0 or C22:1, whilst Stx2c prefers receptors with a C18:0 or C18:1 fatty acid. Because both toxins poorly compete for binding to their preferred receptors, the toxins presumably bind to different but overlapping carbohydrate epitopes, being presented differentially in the membrane environment because of the differences in the membrane anchor of the respective Gb_3_/CD77 molecules [185]. Relative abundance of Gb_3_/CD77 molecules harboring C16 and C22-C24 fatty acids may vary between cells of similar provenience and specialization, as exemplified by the human colon epithelial cell lines Caco-2 and HCT-8 [192], as well as between cells at different stages of differentiation [164].

#### 3.3.4. Receptor-Binding Domains of the Toxins

**Figure 4:**
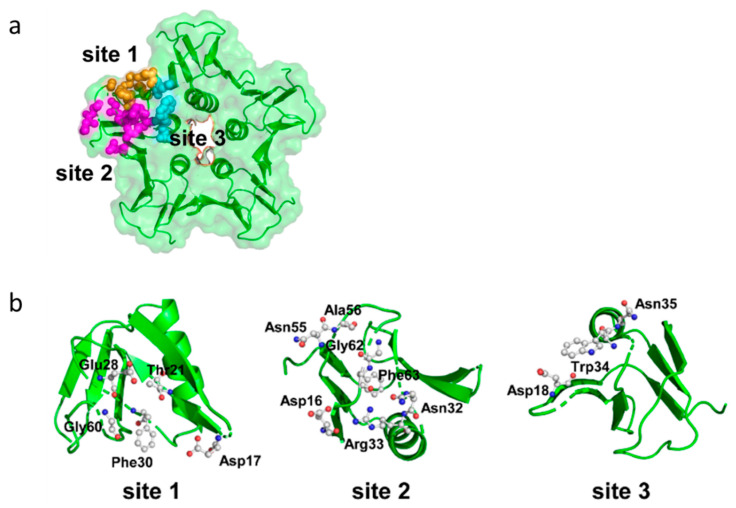
Structural model of Shiga toxin 2 B subunit pentamer holotoxin and Gb_3_/CD77 bindings sites. (**a**) Ribbon diagram of Stx2, planar view on the surface of the B subunit pentamer. The three binding sites per B subunit are exemplarily shown for a single B subunit in orange (site 1), magenta (site 2), and cyan (site 3). (**b**) The amino acids involved in receptor binding (C, gray; N, blue; O, red) are represented by ball-and-stick models colored in green. Reproduced from reference [92]. Elsevier, 2018.

Stxs bind to receptors via the B subunit pentamer [126,193,194]. Upon receptor binding, the A subunit is located on the membrane-far side [77] and, at least for Stx1, is not involved in the binding process [195]. Binding principally depends on amino acids Asp16, Asp17, Arg33, Trp34, Ala43, Lys53, Gly60 of the matured Stx1 B subunit [81,86,87,89,126] as well as on the disulfide bridge between amino acids 4 and 57 [81]. Binding becomes stabilized by the C-terminal end of the B subunit. Deletion of the last two amino acids (Phe68 and Arg69) reduces Stx1 binding, whereas binding is abolished after deletion of the last four amino acids [89]. The tertiary Stx structure was initially believed to be highly conserved with homologous amino acids also mediating binding of Stx2 group toxins [89,95]. However, up to three independent receptor binding sites exist per B monomer, which differ between toxin types (Figure 4).

Receptor binding site I is a pocket formed by two β-sheets belonging to two neighboring subunits in the pentamer. Polar and acidic side chains of Asp16-17 of the β-sheets form hydrogen bonds with polar groups of the carbohydrates in the receptor [84]. Conserved aromatic rings of Phe30 attach to the sugar rings [77,84]. Receptor specificity for Gb_3_/CD77 is mainly determined by Asp18 and Asp17 in Stx1 and Stx2, respectively. In Stx2e, an asparagine residue at this position and amino acids Gln64 and Lys66 presumably interacting with N-acetyl-galactosamine stabilize binding to Gb_4_ [83,126]. Although this receptor binding site is formed by two adjacent monomers, competitive binding studies with Gb_3_/CD77 separated from the membrane environment showed that the Stx1 B subunit and holotoxin had the same affinity for the receptor [75], competing for Gb_3_/CD77 binding in equimolar quantities [126]. This apparent discrepancy may result from only one side of the pocket determining the affinity, while the other only supports binding. Comparison of the amino acid sequence of CD19, a Gb_3_/CD77 binding host cell molecule on B cells (see Section 3.3.5), revealed an approximately 50% identity to Stx. This particularly concerns those amino acids within the B subunits which form the receptor binding site between two B subunits in the pentamer. Of note, most homologous amino acids can be found in the (n + 1) B subunit, suggesting that this side of the receptor binding site is sufficient to define the glycolipid binding specificity, while the opposite side only promotes access to the receptor [196].

Based on cristallographic studies, Lingwood et al. [82], considered site I the most important receptor binding site of Stx1. However, Kitova et al. showed by resonance mass spectrometry that binding of Gb_3_-homologue P^K^ trisaccharide to site I only occurs after saturation of five different binding sites in the Stx1 B subunit pentamer [197]. This receptor binding site II is located within the Stx1 B subunit on the opposite side of Phe30 and is formed by a glycine loop (Gly60-62) and Asn32 and Arg33 [84]. Binding energy between isolated Gb_3_/CD77 and site II is only approximately 50% of the energy of Gb_3_/CD77-site I interactions [84]. However, differences in affinity become significantly smaller in case Gb_3_/CD77 is embedded in a lipid membrane [198], because the immediate environment of the receptor significantly impacts on its binding properties (see Section 4.1.1). The disulfide bond between residues Cys3 and Cys56 in the Stx2 B subunit is conformationally different from the bond between Cys4 and Cys57 in the Stx1 B subunit [80]. Binding of Gb_3_/CD77 to the receptor binding site II of Stx2 would require a conformational change of this disulfide bond in the same way as Stx1, because the carbohydrate chain of the receptor would collide with Ser54 [80]. Site II is preferentially utilized by Stx2c, because this toxin harbors an asparagine residue at the position homologous to Asp17 in the Stx1 B subunit, which is not able to establish hydrogen bonds with the carbohydrate moiety of Gb_3_/CD77 [82,84]. These variations between toxin subtypes are believed to explain the different receptor affinities of Stx1, Stx2, and Stx2c and influence the biological activities of Stxs in tissues [80].

Additionally, a low-affinity receptor binding site III at the N-terminus of the StxB α-helix has been described, at the basis of the B pentamer where the C-terminus of the A_2_ fragment protrudes from the central pore [78,80,199]. However, this binding site appears to be of minor importance for conferring the cytolethal activity of Stx1 [200]. Different from Stx1, the five Trp33 of each subunit in the Stx2 B pentamer have a diverse orientation [80]. In order to bind Gb_3_/CD77 at this site, all five tryptophan residues have to be flexible to such an extent that they may twist to the adjacent asparagine residues in position 34 to generate a suitable binding conformation [80].

#### 3.3.5. Interactions with Physiological (Host) Ligands

CD19 is a 95 kDa protein and a member of the Ig superfamily that is well conserved across species [201]. Its extracellular region consists of three potential domains interlinked with disulfide bonds. Expression of CD19 is the first indication of an hematopoetic cell differentiating into a B cell and only gets lost upon terminal differentiation into a plasma cell. CD19 forms a complex with CD21, CD81, and Leu-13 at the cell surface. Ligand binding to CD19 initiates signal transduction and activation of integrin-dependent adhesion, resulting in proliferation and B cell maturation [196], but also apoptosis [202]. CD19 expression was also found to regulate TLR-4 signaling through p38 mitogen-activated protein kinase (MAPKp38) activation [203]. Amino acid sequences of human CD19 and the Stx1 B subunit are nearly 50% homologous. This particularly concerns extracellular domains of CD19 and amino acids forming the receptor binding site I in the Stx1 B subunit pentamer. Consequently, CD19 is capable of binding to Gb_3_/CD77 on B cell surfaces [196]. Only when complexed with Gb_3_/CD77, CD19 is retrograde transported by the ER-nuclear membrane route [202]. Mutants lacking Gb_3_/CD77 possess CD19 molecules with reduced affinity for Gb_3_/CD77 and are unable to activate integrin-dependent cellular adhesion [196]. It is assumed that Gb_3_/CD77 and CD19 interact on the surface of B cells in a multi-stage process. Initially CD19 is expressed on B cells as an immature protein, devoid of disulfide bonds. Binding of CD19 to Gb_3_/CD77, present on the surface of the same B cell, brings thiol groups in closer proximity, fostering establishment of a disulfide bond and maturation of CD19. Mature CD19 molecules participate in two forms of cell-to-cell adhesion which play an essential role for homing of B cells and establishment of germinal centers in lymphatic organs in vivo. On the one hand, CD19 and Gb_3_/CD77 on neighboring cells interact and allow binding of B cells to each other and to follicular dendritic cells. On the other hand, CD19 binds to Gb_3_/CD77 on the same cell and undergoes conformational changes. These changes are the pre-requisite for signaling from the CD19/CD21/CD81 complex and lead to strong cellular adhesion via integrins. Both types of adhesion anchor B cells in germinal centers. If Gb_3_/CD77, and probably CD19, is down-regulated at the end of B cell differentiation, this adhesion is lost and B cells leave the germinal center. In fact, human B cells only express Gb_3_/CD77 in germinal centers and terminally differentiated plasma cells lack CD19 [196]. If Stx binds to Gb_3_/CD77, the molecules are no longer available for CD19 binding. This may significantly affect human B cell maturation and differentiation by inducing apoptosis, but may also prevent apoptosis by hampering CD19 cross-linking on the cell surface [202].

At protein level, subunit 1 of Interferon-α receptor (IFNAR-1) is homologous to the parts of StxB responsible for pentamer binding to Gb_3_/CD77. This homology particularly exists in human and bovine IFNAR-1 [204]. Even though sequence homology of IFNAR-1 is lower than that of CD19 [196], IFNAR-1 is able to bind Gb_3_/CD77 on cell surfaces [204]. Interactions with Gb_3_/CD77, as well as with Gb_2_ and probably with Gb_4_, induce conformational changes of the receptor, transforming it from the low-affinity to the high-affinity state [87]. In this way, glycolipids modulate IFNAR-1 without affecting its expression. Only binding of IFN to its high-affinity receptor allows exertion of biological effects [204]. Presumably because of its pentameric structure, Stx has a higher affinity to certain isoforms of Gb_3_/CD77 than IFNAR-1 [87]. Incubation of Daudi cells in the presence of Stx1 results in a significantly reduced binding capacity for IFN-α to levels comparable to mutants devoid of Gb_3_/CD77 [204]. These mutants exhibit a markedly lower IFN-dependent activation of cytosolic transcription factors [204], and consequently a lower proliferation inhibiting [205] and anti-viral activity of IFN-α [206]. Accordingly, Stx1 can block the anti-invasive effect of IFN-α on bacterial invasion in HEp-2 cells expressing physiological levels of Gb_3_/CD77 [207] and Stx-producing *Shigella flexneri* bacteria are resistant to the respective effect of IFN [207]. Isolated B subunits are not sufficient to remove bound IFNAR from Gb_3_/CD77 [206]. Binding competition occurs at the cellular surface, not requiring endocytosis of the Stx or its B subunit [206], but inhibition of protein biosynthesis by Stx may synergize with the blockage of IFN [207]. Interestingly, various effects of IFN-α appear to be mediated by IFNAR molecules interacting with different Gb_3_/CD77 isoforms. Gb_3_/CD77 isoforms with long-chain fatty acids primarily exist in the plasma membrane and participate—after interacting with IFNAR-1—in conferring the antiviral activity of IFN-α. Gb_3_/CD77 isoforms with short-chain fatty acids, on the opposite, are preferentially internalized and confer the cytolethal effect of Stx as well as the IFNAR-1-dependent proliferation-inhibiting effects of IFN-α [206].

In silico analyses revealed an amino acid sequence similarity of Stx with the β-chain of human and murine major histocompatibility complex class II (MHC-II) [208]. Different from CD19, the surface expression of MHC-II is unaffected on Gb_3_/CD77-deficient Daudi cell mutants even if the cells possess a decreased total MHC-II protein content. It was assumed that binding of Gb_3_/CD77 to MHC molecules can modify their peptide-binding properties [208].

Beyond a direct functional impact, the existence of Stx-like Gb_3_/CD77 binding sites in CD19, interferon receptor and MHC-II may be of paramount relevance for the course of STEC infections because such homologies may force the immune system to suppress specific responses to Stxs to prevent autoimmunity. In addition to a direct impact of Stxs on human B cells, this may explain why humans usually only develop low antibody titers against the toxins [196,209].

## 4. Shiga Toxins’ Modes of Action

### 4.1. Internalization and Enzymatic Activity

#### 4.1.1. Receptor-Mediated Endocytosis

Stxs were the first ligands recognized to utilize glycolipid receptors for endocytosis via clathrin-coated vesicles [95]. In colonic carcinoma cell lines and primary HUVEC, Toll-like receptor 4 (TLR-4), a pattern recognition receptor for LPS, facilitates Stx binding to cells expressing Gb_3_ [210]. In these experiments, clathrin-dependent Stx1 holotoxin uptake by the epithelial cells was greater than uptake of Stx1 B subunits, suggesting that the A subunit is implicated in TLR-4-aided toxin internalization [211].

Ten to 15 min after binding to Gb_3_/CD77 in lipid rafts [178], receptors become enriched in clathrin-coated membrane pits by lateral movement [187]. The underlying mechanism remains to be elucidated [211] but may involve membrane-mediated mechanisms that drive toxin molecules together [49]. Stx molecules would suppress thermally excited membrane fluctuations not only at the sites at which they bind, but also on the membrane patch between two adjacent toxin molecules, as long as these are not further apart than approximately the size of the toxin itself [212]. Unperturbed fluctuation of the membrane outside this toxin-delineated patch would push the toxin molecules together, even if these were not experiencing a direct attractive force [49]. Furthermore, because Gb_3_/CD77 molecules lack a transmembrane domain, interactions with membrane proteins, like CD19 [202], IFNAR-1 [204], and MHC-II [208], may help to hold back the Stx receptor, cross-linked by bound multivalent toxin, in membrane pits [213]. The high density of certain Gb_3_/CD77 isoforms is believed to further support clustering of bound Stx molecules in lipid rafts [178]. Importantly, binding of the complete AB_5_ Stx holotoxin seems to be specifically required for the ability of high toxin concentrations to induce an increased rate of toxin endocytosis [211]. In A431 and BHK cells, stimulation of Stx endocytosis is not due to an unspecific aggregation of glycosphingolipids in lipid rafts in the plasma membrane, nor is a mere aggregation of Gb_3_ by Stx B subunits sufficient to stimulate Stx internalization. It was suggested that the A subunit of Stx is directly involved in important interactions either to other A subunits, which could possibly cluster the toxins, or to other plasma membrane proteins, which might facilitate toxin internalization like TLR-4 [211]. Alternatively, the A subunit might influence toxin internalization indirectly by affecting the exposure of its associated B subunits. This could change the surface location of the toxin or facilitate interactions with other membrane proteins fostering internalization. Since the interaction of human serum amyloid protein with Stx2 is mediated by both, the A subunit and the B pentamer [214], interactions with soluble proteins may further impact on the binding and internalization process of Stxs to target cells in vivo.

Molecular dynamics simulations suggest that the B subunits induce an increment of negative inward-oriented curvature when interacting with a patch of membrane that contains Gb_3_ receptor molecules [215]. When 13 out of 15 Gb_3_ binding sites per Stx B subunit molecule are occupied, positioning 10 of the Gb_3_ binding pockets at the rim of the B subunits in a location slightly above the normal plane of the membrane, the latter must bend up to reach these sites [215]. Pinching off membrane invaginations enriched for receptor-bound Stx from the plasma membrane likely occurs by fusion of the opposing walls of invaginated tubular endocytic pits [49]. This process may involve the conventional pinchase dynamin [216] and actin as one of the formation triggers [217]. Alternatively, the formation of a highly curved membrane domain sends out a mechanical signal [49], recognized by proteins of the Bin, Amphiphysin, and Rvs (BAR) domain family [218]. Endophilin-A2 was functionally localized on Stx-induced membrane invaginations in relation to the scission reaction [219] and may give rise to a dynein-mediated pulling force.

At the time the toxin is enriched in certain areas of the membrane, Stx can readily be detected in tubular and vacuolar endosomes [187]. After fusion of endosomes with acidic vesicles, vesicles containing 5%–10% of internalized toxin are transported along microtubules [220] to the trans-Golgi network at 37 °C within 60 min [187,213]. The underlying sorting mechanism is determined by the length of the fatty acids in the Gb_3_/CD77 molecules [221,222] and independent of Rab9 [223] or Rab5 (S34N mutant) [224]. Instead, γ-Adaptin [225], SNARE proteins VAMP2, VAMP3, and VAMP8 [226,227], Syntaxin 5 [228], the phosphoinositol-binding clathrin adaptor EpsinR [229], Rab5a, and TRAPPC6B [230] are involved in this cAMP-dependent process [222]. While the receptor returns to the cell surface presumably within minutes [231], the toxin remains in the cell even if it is not translocated into the cytosol [187].

Stx does not contain a KDEL-motif [232], but, via the Golgi apparatus and by a specific retrograde transport route relying on the small GTPase Rab6a [226,233], reaches the endoplasmic reticulum (ER) and the nuclear membrane [234]. Transport involves Syntaxin-16 and Syntaxin-5, EpsinR, Arl1, OCRL, retromer, the tethering complex GARP and the GARP interactor TSSC1, the ARF1 GAP protein AGAP2, GPP130, the ERM proteins Ezrin and Moesin, annexins A1 and A2, and UNC50 (as reviewed in [49]). The route of transport is determined by specific, yet ill-defined properties of Gb_3_/CD77 indicated by the fact that human CD19 becomes retrograde transported to the nuclear membrane only when complexed with Gb_3_/CD77 [202]. For exerting cytotoxic effects, transport of Stxs to the ER is essential, as the indispensable translocation into the cytosol only occurs here [187]. Inhibition of endocytosis by ATP deprivation, inhibition of the cytoskeleton, lowering of intracellular pH or temperatures below 20 °C renders cells resistant to Stxs [114,187,235]. For efficient endocytosis to occur, polarization of cells seems to play a minor role as the toxin was found to be internalized from the apical and baso-lateral side of cells with equal efficacy [109,236].

Interestingly, many toxin-resistant cell lines bind Stx with high affinity [181]. It is assumed that reduced levels of cellular sensitivity are a result of slower internalization, inefficient intracellular processing, a low sensitivity of the protein synthesis machinery, or combinations thereof. Indeed, some cells possess an alternative endocytosis pathway for Stxs, mediated by Gb_3_/CD77 molecules located outside lipid rafts in the cell membrane [178,224]. This pathway is clathrin-independent [224] and much slower than clathrin-dependent endocytosis, due to the ~ 1 min half-life of clathrin-coated pits at the cell surface [237]. Clathrin-independent endocytosis transports Stx to late endosomes in which the toxin is degraded [178]. Stx may be transported efficiently at least to the Golgi apparatus [224], but the respective cells are 1000-fold less sensitive than cells realizing transport to the ER and the nuclear membrane [221]. The considerable variation of eukaryotic cells in the sensitivity to Stx is determined by the lipid moiety of the Gb_3_/CD77 isoforms and its consequence for the intracellular transport route, as well as by the ratio of clathrin-dependent and -independent endocytic processes [238].

#### 4.1.2. Gb_3_/CD77-Independent Endocytosis

In vitro studies showed that human blood cell-derived microvesicles containing Stx undergo endocytosis in human glomerular endothelial cells where microvesicles release their content of Stx within 12 h of entering the cell and the toxin reaches ribosomes within 24 h, leading to cell death by inhibition of protein synthesis [34]. Evidence exists that Stx2-containing microvesicles play a pivotal role in transport and transfer of Stxs in vivo and are even transferred from cell to cell [34], suggesting that Gb_3_-independent targeting of host cells is a yet under-appreciated pathogenic mechanism, presumably with significant implications for the course of STEC-associated diseases. However, this apparently Gb_3_-independent process of toxin uptake requires the presence of Gb_3_ in order for the initial binding of Stx to platelets [33], monocytes [157], and red blood cells [31] to occur.

Human neutrophils are major toxin carrier cells but lack Gb_3_-type Stx receptors. Instead, TLR-4 binds Stx to the neutrophil surface without triggering toxin internalization and intracellular routing [239,240]. Different from human monocytes, in which pronounced cytokine response to Stx depends on Gb_3_, brief incubation (90 min) of human neutrophils with Stx1 results in the release of only minute amounts of proinflammatory mediators [241].

#### 4.1.3. Intracellular Processing in the Target Cell

The enzymatic function of Stxs is associated with an enzymatic cleavage product of the A subunit [242]. By tryptic cleavage at arginine residues 248 or 251, two fragments of 27 and 6 kDa are generated that at first remain connected by a disulfide bound. Upon its reduction, the enzymatically active 27 kDa A_1_ fragment is released [243]. Proteolytic cleavage of Stx A subunit was detected in bacterial lysates as well as within Vero cells [244]. The former finding indicates that Stx, released from STEC by secretion or in outer membrane vesicles [34], may reach the target cells in a pre-activated stage. Furthermore, Stx2d is activated 10- to 1000-fold for Vero cell toxicity by preincubation with intestinal mucus containing elastase, whereas Stx2, Stx2c, Stx2e, and Stx1 are not activatable [64]. The peculiar feature of Stx2d is determined by two amino acids of the A_2_ fragment that represent the only amino acid differences to the non-activatable Stx2c and are cleaved off by elastase. This process requires the presence of the homologous Stx2d B pentamer, suggesting that activation involves B pentamer-dependent cleavage by elastase of the C-terminal two amino acids from the Stx2d A_2_ fragment [64].

In the course of cellular intoxication, reduction of Stx likely occurs in endosomes or the trans-Golgi network. At a pH of 5–6, the Stx A subunit is cleaved by a soluble form of Furin, a calcium-sensitive serine protease with a specificity for Arg-X-Arg/Lys-Arg motifs [245,246]. Alternatively, Stxs may also be cleaved by the cytosolic protease calpain but with low efficacy only [245]. In cell-free systems, the purified Stx1 A_1_ fragment is 3-times more effective than enzymatically pre-treated toxin and 6-times more effective than holotoxin [247]. The reason for this difference in activity is determined by the structure of the A_2_ fragment, in which the methionine in position 260 is positioned in the enzymatically active cleft of the A_1_ fragment blocking the enzymatically active sites of Stx1 and Stx2 holotoxins, while the ribosome binding sites remain exposed to the solvent [248]. The A_1_ fragment only becomes fully active after removal of the A_2_ fragment [88,248]. Inhibition of endosomal fusion with lysosomes and inhibition of proteolytic degradation of endocytosed proteins prevents cells from the cytotoxic effects of Stxs [235]. Further degradation of Stxs does not take place in sensitized MDCK cells even 2 h after the addition of the toxin [109].

#### 4.1.4. Translocation of the A Subunit into the Cytosol

Translocation of the A_1_ fragment into the cytosol is essential for intoxication of cells [104,238] and occurs at the ER. Some reports question that endosomal-lysosomal fusion is required for processing and translocation of the toxin [249]. However, at low pH values in vitro, the Stx1 B subunit undergoes conformational changes [79]. At pH 4.5 marked yet reversible changes at Trp34 take place. This residue is located at the orifice to the central pore formed by the α-helices of the five B subunits in the pentamer. Changes are likely originating from protonation of the aspartate side chains of residues 16 or 18 and the resulting interruption of a salt bridge to the adjacent Arg33. This leads to destabilization of the α-helix‘ N-terminus or interferes with the polarity in the vicinity of the tryptophan. If pH-value further decreases, the α-helix itself becomes subject to conformational changes. In vitro, these alterations might occur even at higher pH-values, if the B subunits are bound to the receptor and associated with the Stx A subunit [79]. Because the amino acids affected by these conformational changes in the B subunit are detrimental for receptor binding and specificity of the holotoxin [86,87,94,126], changes may facilitate release of the holotoxins from the receptor [231].

During transport to the ER, Stx A subunits dissociate from the B subunits, following proteolysis and disulfide bond reduction [8,245,250]. Fragments of the A subunits associate with host ER intraluminal chaperones ERdj3/HEDJ, GRP94 and BiP, followed by translocation across the ER membrane into the cytosol [251,252]. Stxs, like other AB_5_ bacterial toxins, utilize the host cell ER-associated protein degradation machinery to facilitate translocation but A subunits re-fold into their active conformation in the cytosol [253]. In the CT molecule, exerting a similar multimeric structure, α-helices of the five B subunits also form a central pore with a diameter resembling that of the Stx1 B pentamer. This pore is believed to act as a transmembrane channel allowing the A_1_ fragment, supported by the A_2_ fragment, to pass and to reach the cytoplasmic side of the membrane [79]. In the ER membrane, CT is also complexed with Sec61p, which transports newly synthesized proteins into the ER lumen as well as misfolded proteins back into the cytosol for proteasomal degradation during normal cell growth [254].

Compelling evidence also exists that the B subunit itself is translocated into the cytosol: by creating fusion proteins with the B subunit, exogenic antigens can be channeled into the MHC-I antigen presentation pathway [255,256,257].

#### 4.1.5. Inhibition of Protein Biosynthesis by Ribosomal Inactivation

**Figure 5:**
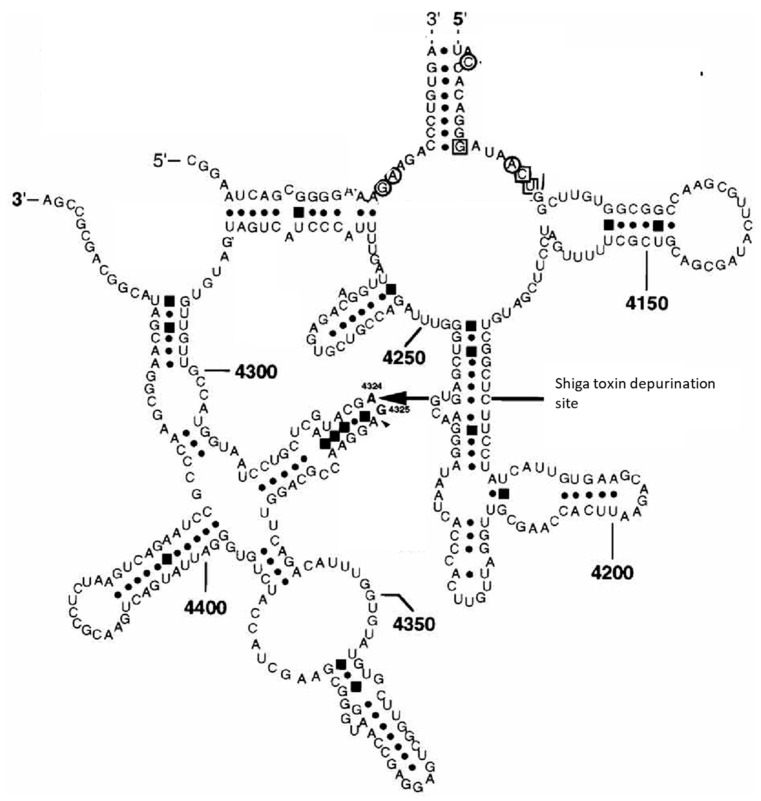
Secondary structure of the 28S rRNA target structure recognized by Shiga toxins. Watson Crick pairs are indicated by dots, non-Watson Crick pairs by quadrants. Reproduced from reference [263]. American Society for Microbiology, 1997.

Stx acts as one of the most potent inhibitors of the protein synthesis machinery in eukaryotic cells [126] by inactivating 60S ribosomal subunits. Notably, the Stx2 A_1_ fragment has a higher affinity for binding to mammalian ribosomes than the Stx1 A_1_ fragment [96]. The nucleotide sequence of ribosomal 28S RNA, as the functionally most important component of the 60S subunit, is highly conserved at positions 4320-4329 in eukaryotic cells [258]. A homologous structure in *E. coli* 23S rRNA binds elongation factors [259]. This sequence forms a hairpin structure [242], with an adenine residue at position 4324 in the loop (Figure 5).

In toxin-treated cells as well as in cell-free systems, this adenine residue is specifically cleaved off by the A_1_ fragment of Stx in a non-phosphorolytic manner [258,260]. This n-glycosidase-activity is independent of several cofactors (NAD, ATP, NADP, NADPH, elongation factors, aminoacyl transferases) [247] and common to all Stxs [95]. The Stx1 A subunit can depurinate ribosomes at physiological pH but depurinate isolated RNA only at an acidic pH [96], indicating that the ribosome creates a microenvironment supportive of enzymatic activity of Stxs. Comparison of amino acid sequences of Stx1 and the plant toxin Ricin yielded three regions of homology at amino acids 51–55, 167–171, and 202–207 of Stx1 [261]. The latter two stretches are located in the enzymatic active cleft of the molecule, the bottom of which is formed by Glu167 and Arg170. The upper part of the side wall is formed by the phenolic rings of tyrosine residues 77 and 114 on the one side and by the rings of Trp203 on the other [262].

**Figure 6:**
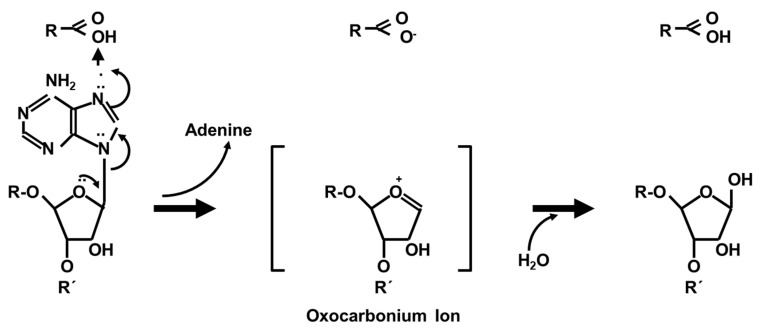
Proposed mechanism of the N-glycosidase activity of Shiga toxins. Reproduced from reference [262]. American Chemical Society, 1992.

The following course of events is believed to take place during the biochemical reaction catalyzed by Stxs (Figure 6) [262]:

1. Arg170 binds the ribose-phosphate backbone of 28S rRNA by forming ionic bonds. Tyrosine 77 and 114 and Trp203 stabilize this binding with their aromatic rings and adjust adenine residue 4324 of the rRNA.
2. Tyr77 transfers a proton to a nitrogen atom in the adenine ring and weakens the bond between C1 of the ribose and N9 of the adenine residue.
3. The protonated adenine dissociates, leaving behind a positively charged oxocarbonium ion in the ribose ring, stabilized by Glu167.
4. Finally, a water molecule attacks the oxocarbonium ion, hydroxylating the ribose and restoring the proton donor Tyr77.

Removal of the adenine residue results in a conformational change in the 28S rRNA and decreases the ribosomal affinity for eukaryotic elongation factor 1 (eEF1), detectable by a significant decrease in eEF1-dependent GTPase activity [264]. As a consequence, eEF1-dependent binding of aminoacyl-tRNA to ribosomes is dramatically impaired [264]. Subsequent steps of the protein biosynthesis (aminoacetylation of tRNA, initiation, peptidyl transferase reaction, translocation) are not directly affected by Stx [264]. Nevertheless, ribosomes become effectively and irreversibly inactivated [247]. This particularly holds for those ribosomes which are bound to mRNA and are biochemically active at the time Stx exerts its effect, because Stxs only target 60S ribosomal subunits if part of a complete ribosome [247]. As a consequence, protein biosynthesis is stopped at the step of elongation, resulting in fixation of the polysomal structure [247], withdrawing the concerned mRNA from interactions with remaining yet intact ribosomes. The reaction rate of the inactivation has been calculated as 40 ribosomes per minute per A_1_ fragment of Stx1 in cell-free systems [247]. For HeLa cells, a ratio of 1000 ribosomes per toxin molecule was deduced [265], a single Ricin molecule reportedly inactivates 1500 ribosomes per minute [266]. The Stx2 A_1_ fragment even exerts a higher affinity for ribosomes, depurinates ribosomes at a higher catalytic rate, and inhibits translation at a significantly higher level than the Stx1 A_1_ fragment in human cells [96].

#### 4.1.6. Nuclear Transport and Intra-Nuclear Effect

Although translocation of the A_1_ fragment into the cytosol occurs at the ER level, the cytolethal effect of Stxs requires retrograde transport of the toxin to the nuclear membrane in some cell systems [221]. In some cells, even accumulation of Stx B subunits within the nucleus has been observed [221]. This transportation pathway is so efficient that DNA fragments can be targeted to the nucleus by means of chimeric proteins made of Stx1 B subunit and DNA-binding proteins [267].

Interestingly, transport of the Stx1 B subunit to the nucleoli even occurs in cells resistant to the protein biosynthesis-inhibiting effect of Stxs [252]. The underlying pathway, described for human macrophages, does not follow the biosynthetic/secretory pathway via the ER [252]. Transport may occur within the cytosol, because elevation of the endosomal pH, essential for translocation of Stx into the cytosol, blocks this transport [252]. This mechanism, however, would require translocation of the B subunit as postulated by Nakagawa et al. [268]. Indeed, the Stx1 B subunit interacts with BiP, an ER-located chaperone, associated with the retrograde transport of proteins to the cytosol [252]. Stx B subunit passively diffuses into the nucleus in permeabilized cells but the transport is ATP-dependent in native cells [252]. After reaching the nucleus, the Stx1 B subunit binds to the nuclear protein B23 (Nucleoplasmin) at equimolar ratios, with both isoforms (B23.1 and B23.2) being recognized equally well [252]. B23 is a multi-functional protein also involved in correct formation of ribosomes. Due to the interaction of the Stx1 B subunit and B23, the Stx holotoxin may be particularly guided to the nucleoli, the generation site of its molecular target [252].

Inactivation of ribosomes is the mechanism the cytolethal activity of Stxs is principally based upon but Stx1 also exerts an adenine-specific N-glycosidase-activity for single-stranded DNA [269]. In vitro, the A_1_ fragment binds to DNA and slides along it until reaching a suitable target structure [269]. Stx1 does not exert a DNAse-activity itself but removal of several adenine residues weakens the sugar-phosphate backbone of the DNA and gives rise to strand breaks [269]. Such was observed in endothelial cells after inhibition of the protein biosynthesis by the ribosomal effects of Stx1 but several hours before induction of DNAses of the apoptosis program [270].

### 4.2. Induction of Eukaryotic Cell Death

#### 4.2.1. Consequences of Protein Biosynthesis Inhibition

Irreversible inhibition of protein biosynthesis by Stxs does not immediately result in the ultimate destruction of the affected cell [271]. In highly sensitive cells, protein biosynthesis starts to decline as early as after 30 min and totally ceases at 45 min. However, cells remain capable of uridine uptake for RNA synthesis for several hours [247,265]. The polysome profile of intoxicated cells remains intact [247]. Cells conduct endocytosis for up to 90 min time with the impact of the toxin and keep their intracellular calcium level constant for up to 120 min. Until then, Stx neither impairs membrane integrity nor oxidative phosphorylation [265]. Only after 4 h, DNA of HeLa cells starts to show signs of fragmentation [272].

Manifestation of functional and subsequent morphological alterations takes several hours in which mechanisms inherited by the targeted cells become activated. Electron microscopy shows circumscribed chromatin masses in the nucleus of Vero cells after 6 h only [271]. The cytosol in the vicinity of the nucleus possesses numerous vacuoles, in part lipid vesicles from blebbing of the nucleus, in part autophagic vacuoles containing membranous material [271]. Inhibition of autophagy prevents cells from lysis, even when deployed at a time the impact of Stx has already terminated cellular protein biosynthesis [271].

Such morphological alterations and degradation of DNA are hallmarks of apoptosis [271]. Although apoptosis represents an active form of cellular death and relies on intact protein biosynthesis in many cells [273], several translation inhibitors may induce apoptosis probably due to an accumulating shortage of proteinaceous apoptosis inhibitors and initiation of the genetic program and cell death [274]. In fact, Stx1- and Stx2-induced apoptosis in human endothelial cells is preceded by a significantly lowered expression of Mcl-1, a member of the anti-apoptotic Bcl-2 family [275]. DNA fragmentation following impact of Stxs cannot be observed in every cell system and apoptosis inhibitors may not always prevent cells from lysis [271]. Nevertheless, inhibition of protein biosynthesis by Stxs is an important co-factor for the induction of apoptosis, if cells have become sensitized by another stimulus before [276]. This stimulus may be Stx itself, implying that the toxins may induce apoptosis, independent of their protein biosynthesis-inhibitory action. In addition, Stx1 sensitizes human endothelial cells to LPS-induced apoptosis by inhibition of the expression of the anti-apoptotic protein FLIP, a caspase-8 inhibitor [140]. Caspase-8-mediated cleavage of Bid and relocalization of its cleavage fragment tBid to the mitochondria is necessary for Stx1-mediated apoptosis in Burkitt’s lymphoma cells [277].

Cells do not exhibit morphological alterations visible by light microscopy for the first 12 h [247] but then begin to loose membrane integrity [247,271]. Stx1 irreversibly arrests HCT116 cells in the S phase within 24 h and only prolonged incubation triggers DNA fragmentation. Concomitant to the activation of the S phase checkpoint, levels increase of mRNA and proteins of growth arrest and DNA damage-inducible gene family, i.e., GADD34, GADD45a, and GADD45b but not of key cell cycle related proteins such as CDK2, CDK4, p21, p27, and p53 [113]. Less sensitive cells, e.g., confluent endothelial cells without cytokine stimulation, only exhibit a 40% decline in protein synthesis and cells remain fully viable for up to 48 h [110]. These endothelial cells represent the main target for Stxs in humans and piglets in vitro, suggesting that sublethal damage of cells rather than total destruction may be of utmost importance in the pathogenesis of Stx-mediated diseases [110].

#### 4.2.2. Direct Activation of the Apoptosis Program

**Figure 7:**
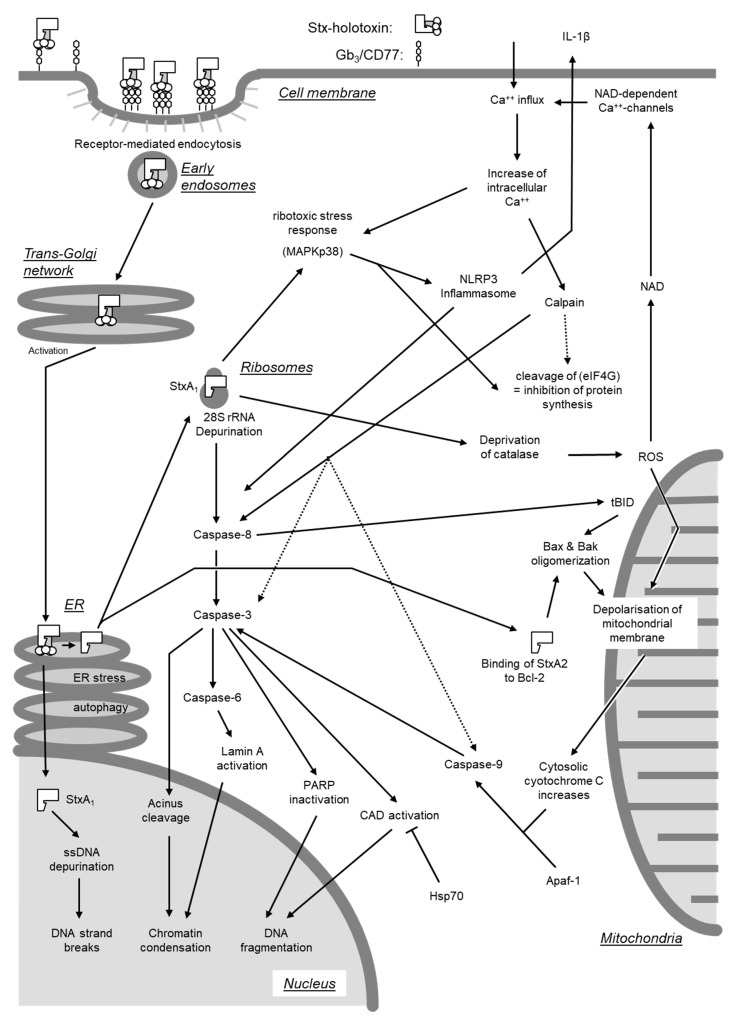
Graphical representation of selected intracellular effects and induction of apoptosis in mammalian cells following internalization of Shiga toxin holotoxins (Stx). Upon binding to the Gb_3_/CD77 receptor, Stxs undergo receptor-mediated endocytosis and incorporation into the early endosomal pathway. Along the microtubule, the toxin reaches the trans-Golgi network and, by further retrograde transport, the endoplasmic reticulum (ER) and eventually the nucleus. In the ER, the A subunit of the holotoxin is cleaved and the A_1_ fragment is translocated into the cytosol. At the ribosomes, A_1_ fragments lead to depurination of 28S rRNA subsequently causing decreased ribosomal binding affinity to e(EF1), irreversible inactivation of ribosomes, and inhibition of protein synthesis (ribotoxic stress response). In the ER, A_1_ fragments induce the unfolded protein response (UPR) indicated by activation of ER stress markers (IRE-1, PERK, not shown). C/EBP homologous protein is induced, which acts pro-apoptotic as Bcl-2 is inhibited (not shown). The ribotoxic stress response is linked to NLRP3 inflammasome generation and IL-1β formation. Via nicotinamide dinucleotide (NAD), reactive oxygen species (ROS) initiate an increased Ca^++^ influx into the cytosol by opening NAD-dependent Ca^++^ channels. Intracellular, the calcium-dependent cysteine protease calpain is activated. Calpain can activate caspase-8 directly, yet many other targets of calpains are known like actin, caspases-3 and -9 (activation), and cleavage of the eukaryotic initiation factor 4G (eIF4G), resulting in inhibition of protein translation. Starting from caspase-8, a cascade of events is initiated encompassing activation of caspase-3 and caspase-6. The subsequent activation of signaling molecules/caspases is also subject to positive feedback loops (e.g., caspase-6 activating caspase-8, not depicted) and linked to the mitochondrial apoptotic pathway (caspase-9). The integrity of DNA is targeted via activation (lamin A, acinus, CAD) or inactivation of factors (PARP), or depurination by Stx A_1_ fragment. ROS and tBid cleavage are linked to depolarization of the mitochondrial membrane and Apaf-1/cytosolic cytochrome C-mediated caspase-9 activation. Stress-induced proteins like HSP70 inhibit activation of CAD. Dotted lines indicate fundamental cellular biology mechanisms not yet specifically linked to Stxs; for details and references see text.

Beyond inhibition of protein synthesis, Stxs have also been found to directly induce apoptosis by activating either of two signaling pathways [278]. One pathway originates from cross-linking of Gb_3_/CD77 on cellular surfaces (see Section 4.2.3.), whereas the second requires internalization of the enzymatically active A subunit into the cytosol (Figure 7).

Depurination of 28S rRNA at translationally active ribosomes by the Stx A subunit leads to structural alterations in critical regions of the rRNA and functional impairment during translation [263]. These deviations result in the activation of stress-activated protein kinases (SAPK/JNK) and induce a ribotoxic stress response (see Section 4.3) [263], induction of the expression of various chemokine genes (see Section 4.5), and caspase activation [279]. Accordingly, inhibitors of the MAPKp38 protect cells from Stx-induced cell death [279,280], despite the fact that inhibition of certain MAPK like ERK may enhance caspase-3 activation [281]. Even though the absence of inflammatory signs is one hallmark of apoptosis, the Stx-induced signaling pathways leading to the expression of proinflammatory genes and to apoptosis are closely interconnected [279].

Activation of caspase-8 is of pivotal importance for induction of apoptosis in Burkitt’s lymphoma cells [278], Hep2 [282], and HeLa cells [272]. Activated caspase-8 cleaves and activates the central effector caspase-3 [272,278,283]. Caspase-3, in turn, cleaves the nuclear factor acinus [284] and caspase-6 [272,285], which cleaves and activates lamin A [286]. In addition, a positive feedback loop exists with caspase-6 directly activating caspase-8 [287]. Activation of acinus and lamin A results in chromatin condensation [284] and disruption of the internal nuclear structure [286]. Caspase-3 inactivates an inhibitor of caspase-dependent DNases (CAD) [272] as well as poly(ADP-Ribose)-polymerase PARP [282], a DNA repair enzyme. Activation of CAD in conjunction with inhibition of DNA repair enzymes results in DNA fragmentation by inter-nucleosomal cleavage. Furthermore, single-stranded DNA becomes more prone to undergo strand breaks following depurination catalyzed by StxA in the nucleus [269]. Treatment with curcumin, inducing expression of heat shock protein Hsp-70, prevents DNA fragmentation and protects cells from the cytolethal effect of Stxs [288].

Stxs also induce activation of caspase-2 and -10 in THP-1 cells [285] and caspase-7 in Burkitt’s lymphoma cells [283].

Activation of caspase-8-dependent signaling pathways is of prime importance for the induction of apoptosis by Stx holotoxins in Burkitt’s lymphoma cells [278]. Nevertheless, in Stx-treated HeLa and Hep2 cells, caspase-8 also cleaves BID, a pro-apoptotic member of the Bcl-2 family located in the outer mitochondrial membrane [272,282]. Activated BID (truncated BID, tBID) promotes oligomerization of the pro-apoptotic proteins Bax and Bak. The subsequent increase in the permeability of the mitochondrial membrane leads to the collapse of the membrane potential and release of cytochrome C into the cytosol [272]. Cytosolic cytochrome C forms complexes with Apaf-1 (apoptotic protease activating factor) and activates caspase-9 [272,282,285,289]. Activation of caspase-9 promotes cleavage and activation of caspase-3 induced by caspase-8 [272,285].

Of note, the polypeptide chain of the A subunit of Stx2 contains a sequence motif (NWGRI, amino acid residues 223–227) homologous to the BH1-Domain of the anti-apoptotic Bcl-2 [290]. This domain is indispensable for the anti-apoptotic effect of Bcl-2 and normally is involved in the interaction with other Bcl-2 molecules and with Bax and Bak. By utilizing the BH1-homologous domain, StxA2, after translocation to the mitochondria, may form complexes with Bcl-2, removes Bax and Bak and also induces their oligomerization [290]. Although Stx1 also possesses a sequence (NWGRL, amino acid residues 234–238) with high similarity to the Bcl-2 BH1-domain, only Stx2 but not Stx1, interacts with Bcl-2 via NWGRI [290]. This may explain why the mitochondrial signaling pathway resulting in caspase-9 activation does not play a role in the effect of Stx1 on Burkitt’s lymphoma cells [283]. Ectopic expression of Bcl-2 prevents Gb_3_/CD77-mediated apoptosis by the Stx1 B subunit (see Section 4.2.3), but not the effect of the corresponding holotoxin [291]. As recently reviewed by Lee et al. [8], enhanced protein and mRNA expression of Bcl-2 is associated with protection from apoptosis induced by Stx1 in toxin-resistant macrophage-like cells under ER stress, while Bcl-2 expression is decreased in toxin-sensitive monocytic cells, leading to rapid apoptosis in the presence of the toxin [292]. Furthermore, amino acid Ser70 of Bcl-2 is phosphorylated, and the protein fails to translocate to mitochondria following Stx1 treatment of macrophage-like cells, whereas phosphorylation of Bcl-2 at Ser70 is significantly reduced in toxin-treated monocytic cells [292]. Although mature macrophage-like THP-1 cells are relatively resistant to the rapid induction of apoptosis by Stxs, downstream signaling through the apoptosis-inducing receptor-ligand pair DR5-TRAIL during ER stress contributes to delayed apoptosis detected in Stx1-treated macrophage-like THP-1 cells [293].

Oxidative stress following an intracellular increase of reactive oxygen species (ROS) causes an increase in permeability of the mitochondrial membrane [289]. Upon intoxication of cells by Stx, this stress may be the consequence of a reduced expression of cellular catalase [280,294]. When acting in concert with LPS, Stx2 activates caspase-4, gasdermin D, and the NLRP3 inflammasome in human THP-1 macrophages in a Gb_3_-dependent manner [295]. Resulting Stx2/LPS mediated IL-1β secretion and the inflammatory form of apoptosis (“pyroptosis”) are dependent on mitochondrial ROS, downstream of the non-canonical caspase-4 inflammasome and cleaved gasdermin D, which is enriched at the mitochondria [295]. In addition to their mitochondrial effect, ROS induce an increase in cytosolic NAD, thereby triggering an opening of NAD-activated Ca^++^ channels at the cellular membrane [296]. Uncontrolled influx of Ca^++^ ions in Stx-treated cells results in phosphorylation of MAPKp38 [280], creating a positive feedback loop to the ribotoxic stress response. At least in some cell systems, the Ca^++^-influx appears to play a major role as Ca^++^ channel blocking agents like verapamil protect Vero and HeLa cells from the cytolethal effect of Stxs [249].

#### 4.2.3. Activation of Gb_3_/CD77-Dependent Signaling Pathways

**Figure 8:**
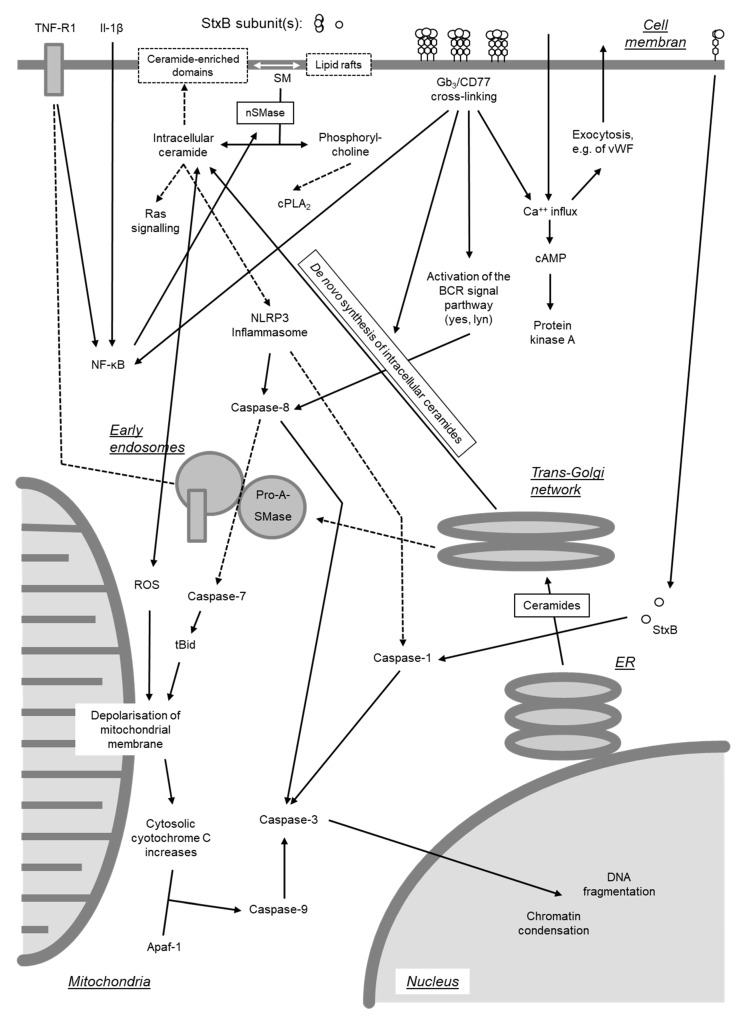
Graphical representation of effects in mammalian cells following binding of Shiga toxin (Stx) B subunits to cell surface receptors. Binding to Gb_3_/CD77 cross-links several receptor molecules and thereby activates signaling cascades as well as internalization of the complex. In human B cells, binding of Stx to Gb_3_/CD77 in lipid rafts induces an enrichment of Src kinases and initiation of the apoptosis-inducing signaling cascade associated with the B cell receptor. Activation of this cascade synergizes with apoptosis induction resulting from the cytosolic effects of Stx holotoxins. Cross-linking of Gb_3_/CD77 also causes a massive influx of extracellular Ca^++^ ions followed by an increase of intracellular cAMP concentrations, activation of protein kinase A (PKA) and, e.g., acute von Willebrandt factor secretion from human endothelial cells. Stxs also likely impact on the sphingomyelinase-ceramide pathway. Sphingomyelinase (SMase) catalyzes sphingomyelin (SM) hydrolysis. Phosphorylcholine and intracellular ceramide activate cytosolic phospholipase A_2_ (cPLA_2_). Intracellular ceramide generates ceramide-enriched regions in the membrane, serves as second messenger (signaling) and activates inflammasomes. Different pathways are affected by ceramide like the Ras and PLA_2_ signaling pathway. Stx binds to the Gb_3_/CD77 receptor, activates the first two enzymes of the Gb_3_ synthesis pathway and induces their transcription. The de novo synthesis is complex and occurs at the trans-Golgi network and the ER. Intermediates (ceramides, lactosylceramide) induce reactive oxygen species (ROS) at the mitochondria, which are linked to the depolarization of mitochondrial membranes and the release of cytochrome C. Furthermore, ROS can activate SMases. IL-1β and receptors, which undergo crosstalk with Gb_3_/CD77-mediated signaling pathways, activate NF-κB. Vesicles containing lysosomal SMases fuse with early endosomes containing TNF receptor (TNF-R1). Subsequently, caspase-7 is activated by caspase-8, leading to activation of SMase and tBid cleavage [307]. The mechanism has not yet confirmed to be implicated in Stx-mediated effects but it is reasonable to assume a possible link between SMase function and crosstalk of TNF-α, Gb_3_/CD77 receptor, and apoptosis, which are proven to be associated with Stx. Gb_3_/CD77-bound B subunit can also be translocated into the cytosol, trigger caspase-1 and -3 activation and directly initiate apoptosis. Pathways depicted in this figure are interconnected at several levels (inflammasome formation and IL-1β secretion; caspase activation; tBid; ROS) with pathways presented in Figure 7. Dotted lines indicate fundamental cellular biology mechanisms not yet specifically linked to Stxs; for details and further references see text.

Several tumor cell lines resist apoptosis induction by isolated B subunit of Stxs [272,285,297]. By contrast, cross-linking of Gb_3_/CD77 at the cell surface by anti-CD77 antibodies or binding of StxB stimulates intracellular signals and apoptosis in Burkitts’ lymphoma cells [278,298,299,300], as well as in the human renal tubular cell line ACHN [179,298]. In the absence of the A subunit, the B subunits of both Stx1 and Stx2 bind to the glycolipids, but the more stable B pentamer formed by Stx1 binds better than the less stable pentamer of Stx2 [186], implying that both toxins are unequally able to initiate this signaling pathway (Figure 8).

In sensitive cells, Gb_3_/CD77 is located in lipid rafts in spatial proximity to Src kinases Yes and Lyn as well as Syk [179,298,301]. Ten minutes after binding of Stx, tyrosine residues of raft proteins become hyper-phosphorylated. Binding of Stx to Gb_3_/CD77 initially induces an enrichment of Yes and Lyn in the lipid rafts. Following activation, Yes and Lyn are removed from the lipid rafts but remain associated with the cellular membrane and do not follow the internalization of the Stx-Gb_3_/CD77 complexes [179,298]. In B cells like Burkitt’s lymphoma cells, the apoptosis-inducing signaling cascade originating from Gb_3_/CD77 is closely linked to the signaling pathway originating from the B cell receptor [301]. Activation of the signaling cascade by surface binding of Stxs synergizes with the apoptosis induction, resulting from the cytosolic effects of Stx holotoxins [298].

As early as after 30 s after cross-linking of Gb_3_/CD77, a massive influx of extracellular Ca^++^ ions can be observed, reaching its maximum after 120 s and followed by an increase of intracellular cAMP concentrations and activation of protein kinase A (PKA) within minutes [300]. Interestingly the Stx B subunits induce acute von Willebrandt factor secretion from human umbilical vein endothelial cells within 30 s via PKA (Stx2 B subunit) or PKCα (Stx1 B subunit), thereby eliciting rapid cellular effects themselves [302].

After 30 min, the cellular content of cytosolic ceramide increases [300]. Agonists like TNF-α or IL-1β can cause the release of ceramide from sphingomyelin by activating an endogenous sphingomyelinase, whereas uropathogenic *E. coli*, capable of Gb_3_/CD77 binding via their P-fimbriae, only induce a minor increase in sphingomyelinase activity in epithelial cells (A498) and no detectable hydrolysis of sphingomyelin [303]. An increase of cytosolic ceramide in Stx-treated Burkitt’s lymphoma cells also is not accompanied by a decrease in sphingomyelins [300]. The cellular content of Gb_3_ remains constant under these conditions, arguing against a degradation of the Stx receptor after ligand binding as source [300]. The increase in ceramide content may be the result of ceramide synthase activation as observed after stimulation of bovine γδT cells via the WC1 antigen [304].

Cytosolic ceramide is part of a signaling pathway particularly inducing apoptosis but also modulating cellular growth and differentiation and stimulating cytokine secretion [303]. Elevated intracellular ceramide levels, as inducible by bacterial sphingomyelinase treatment of human endothelial cells, induces increased expression of enzymes of the Gb_3_/CD77 synthesis pathway and increased synthesis and surface expression of Gb_3_/CD77 [141]. Because of the resulting sensitization of cells to Stx [141], it is tempting to speculate that in cells exhibiting at least some degree of Stx responsiveness, binding of a low number of Stx molecules to Gb_3_/CD77 is sufficient to initiate a positive feedback loop comprising of increased receptor expression and subsequent increased toxin uptake. An increased synthesis rate of Gb_3_/CD77 also gives rise to elevated cellular concentrations of the Gb_3_/CD77 precursor lactosylceramide [141].

Intracellular ceramide was also identified as a TLR-4 agonist and putative signaling intermediate between glycosphingolipid receptors and TLR-4 [305]. Binding of microbial ligands to such receptors like P fimbriae or the B subunit of Stxs increases the levels of ceramide and triggers a TLR-4-dependent response in epithelial cells. This presumptive crossing of signaling pathways is of particular importance as TLR-4 facilitates binding of Stxs to epithelial and endothelial cells co-expressing Gb_3_/CD77 [210]. Furthermore, CD19, a Gb_3_/CD77 binding molecule, was found to regulate TLR-4 signaling through MAPKp38 activation [203] pointing to a complex network of signaling pathways Stxs can interfere with.

Ceramide and lactosylceramide both induce the generation of ROS in mitochondria [289,306] and thereby activate the mitochondrial signaling pathway of apoptosis induction, which also plays a central role in the Gb_3_/CD77-dependent signaling pathway [278,291]. Cross-linking of Gb_3_/CD77 on Burkitt’s lymphoma cells leads to activation of caspase-8 [283], indicating that Gb_3_/CD77 binding also can activate the extrinsic, caspase-dependent apoptosis pathway. Interestingly, even the Stx1 B subunit, not inheriting any proteolytic activity, can trigger caspase-1 and -3 activation and initiate apoptosis when artificially expressed inside eukaryotic cells [268].

### 4.3. Ribotoxic Stress Response

The ribotoxic stress response resulting from depurination of 28S rRNA [263] is not specific for StxA but can also be induced by other toxins like Ricin or α-Sarcin which modify the rRNA hairpin structure at positions 4320–4329 [263]. In case ribosomes are biochemically active at the time of impact of the toxins, resulting structural alterations in the peptidyltransferase center of the ribosomes activate small GTP-binding proteins, which in turn activate the protein kinase cascade, which, via MEKK1 and SEK1/MKK4, ends in the activation of stress-activated protein kinases SAPK/JNK1 [263]. The mitogen-activated protein kinase kinase kinase (MAP3K) that transduces the signal from intoxicated ribosomes to activate SAP kinases was identified as a zipper sterile-alpha-motif kinase (ZAK) [308]. Depurination of rRNA at position A4324 is detectable as early as 15 min after addition of Ricin to cell cultures [263]. This is followed after 30 min by sustained phosphorylation of SEK1/MKK4 and activation of SAPK/JNK1 which lasts for hours [263]. Activation of this cascade is independent of protein biosynthesis inhibition and also occurs under conditions in which protein translation is only marginally affected [263]. This may explain why cells, even after intoxication by Ricin or Stxs, still are capable of responding with an increased transcription of the immediate early genes *c-fos* and *c-jun*, which becomes apparent after 60 min [263,279,309]. In some cells, the increased transcription of *c-fos* and *c-jun* appears to be the consequence of MAPKp38 activation [279,309,310]. Stx-induced *c-jun* transcription increases concentrations of the c-Jun protein which is particularly present in its phosphorylated form [311]. Remarkably, the increase in phosphorylated c-Jun is accompanied by an increased expression of *mpk-1* (mitogen-activated protein kinase phosphatase 1), even though this phosphatase can inactivate JNK as well as MAPKp38 [311].

In addition to the SAPK/JNK1 and MAPKp38 signaling pathways, Stx also stimulates PKC activity [312]. Stx-treated human monocytes exhibit an increased activity of kinases, which are located in the signaling pathway downstream of atypical isoforms of PKC and are regulated by extracellular signals (extracellular signal-regulated kinases; ERK) [313]. This implies that Stx-induced PKC activity is realized by atypical PKC isoforms activation of which neither requires calcium nor lipids and which, e.g., are involved in LPS signaling [312].

Stxs activate an autoregulatory signaling loop since inactivation of ERK, JNK, or MAPKp38 in the presence of Stx1 results in differential regulation of TNF-α, IL-1β, IL-8, GRO-β, MIP-1α, and MIP-1β. THP-1 cells exposed to Stx1 upregulate the expression of selected dual-specificity phosphatases (DUSPs), enzymes that dephosphorylate and inactivate MAPKs in mammalian cells [314]. DUSP1 inhibition by triptolide showed that ERK and MAPKp38 phosphorylation is regulated by DUSP1, while JNK phosphorylation is not. In turn, inhibition of MAPKp38 signaling blocked the ability of Stx1 to induce DUSP1 mRNA expression [314]. In addition, signaling through the PI3K/Akt/mTOR pathway was shown to initially activate proinflammatory cytokine expression via the phosphorylation of the eukaryotic translation initiation factor 4E-BP, which is later on down-regulated through the inactivation of the positive regulator glycogen synthase kinase (GSK)-3 [315], suggesting that the activation of cytokine signaling by Stxs ultimately downregulates the proinflammatory cytokine expression.

### 4.4. Endoplasmic Reticulum Stress and Autophagy

It was assumed that bacterial toxins of the AB_5_ type highjack the ER-associated protein degradation pathway to enter target cells by presenting themselves as misfolded proteins [253]. As recently reviewed by Lee et al. [8], Stx1 is capable of inducing ER stress and activating three proximal unfolded protein response (UPR) effectors, PERK, IRE1, and ATF6, involved in the immediate detection of unfolded proteins [316]. Stx1 and Stx2 differentially activate non-overlapping sensors of ER stress rendering the intoxicated cells susceptible to the cytotoxic action of Stxs with significant cleavage of PARP, an enzyme primarily involved in DNA repair and induction of programmed cell death [317]. In Caco-2 cells exposed to Stx2, ER stress promotes autophagic cell death preceding apoptosis [318]. However, induction of autophagy by Stxs has different outcomes depending on the toxin-sensitive or toxin-resistant phenotype of the cells [8]. In toxin-resistant primary human macrophages, Stxs are translocated to lysosomes, and autophagy is induced in the absence of calpain and caspase activation, as well as Atg5 and Beclin-1 cleavage [319]. In toxin-sensitive cells, Stxs are translocated to the ER, the ER stress response is activated and autophagy is induced in association with the activation of calpains and caspase-8 and -3, as well as the cleavage of Atg5 and Beclin-1. Treatment of intestinal epithelial cells with Stx2 initiates autophagic cell death via the ER stress pathway and by triggering pseudokinase TRIB3-mediated DDIT3 expression and AKT1 dephosphorylation [318].

### 4.5. Induction of Proteinaceous (Inflammatory) Mediators

Involvement of signaling pathways as well as the fact that Stx-dependent activation of SAPK/JNK1 and MAPKp38 can be observed in human monocytes [310], indicate that Stxs may induce the expression of proteinaceous mediators even independent of the toxins’ ribotoxic activity. In favor of this notion, the increase in intracellular ceramide concentration—as e.g., observed after Stx1 binding to Gb_3_/CD77 in the absence of the toxins‘ enzymatic activity [300]—has already been associated with an increased cytokine gene expression [303]. Induction of caspase-1 by intracytoplasmic expression of Stx1 B subunit reportedly results in increased synthesis of IL-1β and TNF-α [268]. On the contrary, several studies failed to detect induction of genes following exogenous addition of isolated Stx B subunits or enzymatically inactive holotoxins to cell cultures [279,311,320,321,322,323,324]. Relative to Stx1, Stx2 significantly causes increased expression of GRO, G-CSF, IL-1β, IL-8, and TNF-α in macrophage-like THP-1 cells, which is not due to a difference in cytotoxicity [324].

Activation of inflammatory mediator gene expression by Stxs may also be a consequence of translocation of the transcription activator NF-κB. NF-κB binding sites are present in the promotor regions of many proinflammatory genes. Sakiri et al. [321] showed for THP-1 cells that the Stx1-induced elevation of TNF-α specific mRNA is preceded by the nuclear translocation of NF-κB and AP-1 (activator protein-1) and by the degradation of the NF-κB inhibitor IκBα. In ACHN cells, Stx2 induces increased *TNF-*α transcription via activation of cAMP-dependent PKA, MAPKp38, and NF-κB [325]. In Vero and T84 cells, these effects cannot be induced by purified toxin, but only in combination with a non-identified factor [310] or after infection of the cells with a STEC strain [326]. It is assumed that translocation of NF-κB in the course of the STEC infection is mainly induced by flagellin via TLR-5, as *stx*- and *eae*-negative *E. coli*-strains but not purified flagellin, fail to induce this effect [327].

As a consequence of Stx acting on ribosomes, the intracellular content of mRNA specific for primary response genes is increased in several cell systems. Induction of the genes is refractory to translational blockade. Stxs particularly promote mRNA induction by other agonists, a phenomenon known as super-induction [320,328]. Although other protein biosynthesis inhibitors like cycloheximide increase the cellular content of certain mRNA species by induction of transcriptional activators, as well as by stabilizing mRNA, the latter mechanism seems to be of prime importance for the effects of Stxs [328].

In the human renal proximal tubular cell line HK-2, Stx1 treatment elicits a modest and delayed increase in TNF-α mRNA expression, peaking at 6-fold increase 4 h after toxin exposure [317]. In contrast, Stx2 treatment induces a rapid increase in TNF-α mRNA (200-fold) detected 15 min after toxin exposure. TNF-α mRNA levels then decline by 30 min and begin to increase to levels 100-fold higher than those in control cells by 120 min [317].

In spite of the onset of protein synthesis inhibition after a few hours in less sensitive cells, the elevated total amounts of specific mRNAs give rise to increased neosynthesis of the encoded proteins, which can be detected after 12 h and lasts up to 24 h [328]. The degree of general inhibition of protein synthesis in the cells is negatively correlated with the translational activity for certain proteins [309]. Nevertheless, a massive increase in mRNA is often associated with an only minor increase in the respective protein [309,322,329], and interpreted as a consequence of partial translational blockage [309,322]. It heavily depends on the type and source of cell, which of the aforementioned effects prevail, and consequently what mediators are increasingly expressed and released by cells upon contact to Stxs (Table 3).

Different from other mediators like IL-8 and GRO-β, processing and release of biologically active IL-1β requires the formation of the NLRP3 inflammasome. Indeed, caspase-1 activation, reaching maximal levels at 4 h after treatment of cells with Stx1, correlates with the processing of inactive pro-IL-1β into mature IL-1β [330]. This Stx-mediated inflammasome activation is dependent on the toxin receptor Gb_3_ and enzymatically functional Stxs [330].

**Table 3:** Overview of cytokines and chemokines induced by Shiga toxins or their subunits in cells of varying type and origin.

Cell Type/Line	Species	Toxin	Induction of		Reference
			mRNA ^1^	Protein ^1^	
**Intestinal epithelial cells**					
HCT-8	Human	Stx1	IL-8, GRO-α, GRO-β, GRO-γ, ENA-78	IL-8, GRO-α	[328]
		Stx1 + 2	IL-8	IL-8	[309]
Caco-2	Human	Stx1 + 2	IL-8, MCP-1, MIP-1α, MIP-1β, TNF-α	IL-8	[322]
**Renal epithelial cells**					
Glomerulum, primary	Human	Stx1	IL-1, IL-6, TNF-α	IL-1, IL-6, TNF-α	[331]
Tubulus, primary	Human	Stx1	IL-1, IL-6, TNF-α	IL-1, TNF-α	[332]
HK-2 (proximal tubulus)	Human	Stx1	IL-1β, TNF-α		[317]
		Stx2	IL-1β, IL-8, TNF-α, MIP-1α, MIP-1β	MIP-1α, MIP-1β	[317]
**Endothelial cells**					
Brain, primary	Human	Stx1	IL-1β, IL-6, TNF-α	IL-6, IL-8	[333]
Aortic, primary	Bovine	Stx1 (+2)	Preproendothelin-1	Endothelin	[139]
Umbilical vein, primary	Human	Stx1	IL-8, GRO-α, GRO-β, GRO-γ, TNF-α	IL-6, IL-8, GRO-α, MCP-1	[334]
		Stx2	IL-8, GRO-α, GRO-β, GRO-γ, IL-6, IL-16, MCP-1, TNF-α	IL-6, IL-8, GM-CSF, GRO-α, MCP-1	[334]
**Fibrocytes/-blasts**					
NIH3T3	Mouse	StxB1 ^2^	IL-1β, TNF-α	IL-1β, TNF-α	[268]
**Monocyte/Macrophage-like**					
THP-1, differentiated	Human	Stx1 + 2	TNF-α	TNF-α	[312]
THP-1, differentiated	Human	Stx1	IL-1β, TNF-α	IL-1β	[320]
THP-1, differentiated	Human	Stx1	IL-8, MIP-1α, MIP-1α, GRO-α, IL-1α, TNF-α	IL-8, MIP-1α, MIP-1β, GRO-α	[329]
THP-1, differentiated	Human	Stx1 + 2		GRO, G-CSF, IL-1β, IL-8, TNF-α	[324]
THP-1, differentiated	Human	Stx1		TNF-α	[200]
THP-1, un-differentiated	Human	Stx1		IL-1β, TNF-α	[115]
THP-1	Human	Stx1	TNF-α	TNF-α	[321]
Peripheral blood, primary	Human	Stx1 + 2		GM-CSF, TNF-α	[313]
Peripheral blood, primary	Human	Stx1 + 2	IL-8, IL-1β, TNF-α	IL-8, IL-1β, TNF-α	[335,336]
Peripheral blood, primary	Human	Stx1		IL-1β, TNF-α	[115]
Peripheral blood, primary	Human	Stx1		IL-1β, TNF-α, IL-6, G-CSF, IL-8, CCL2, CCL4	[241]
Peripheral blood, primary	Bovine	Stx1	IL-4, IL-6, IL-10, IFN-γ, TNF-α, IL-8 and GRO-α		[160]
Peripheral blood, primary, non-adherent	Human	Stx1	IL-6	IL-8, IL-1β, IL-6, TNF-α	[157]
Colonic mucosal macrophages, primary	Bovine	Stx1	IL-8, GRO-α, MCP-1, RANTES, IL-10		[133]
Peritoneal exudate	Mouse	Stx2		TNF-α	[337]
Peritoneal exudate	Mouse	Stx1 + 2	TNF-α	IL-1β, IL-6, TNF-α	[159]
Mesangial cells, primary	Human	Stx1	MCP-1		[338]
**Neutrophils**					
Peripheral blood, primary	Human	Stx1		IL-8, CCL4, G-CSF, TNF-α, IL-1β	[241]
**Lymphocytes**					
Ileal intraepithelial	Bovine	Stx1	IL-4		[339]

^1^ Discrepancies in the lists of mediators at mRNA and at protein level principally result from the fact that not all cited studies have quantified the respective mediators at mRNA and at protein level; ^2^ Stx1 B subunit.

### 4.6. Induction of Arachidonic Metabolite Synthesis

The discovery of Zhang et al. [336], that Stx-induced TNF-α expression in human monocytes can be counteracted by treatment of the cells with a synthetic antagonist of platelet-activating factor PAF, points to the existence of yet another Stx-inducible signaling pathway. Indeed, PAF treatment of monocytes together with LPS results in an activation of ceramide synthetase and an increase in intracellular ceramide levels [340]. The latter can also be observed after Stx treatment of Burkitt’s lymphoma cells [300]. The B subunit of CT accelerates cellular synthesis of arachidonic acid metabolites [341], implying that a signal originating from glycolipid receptors in lipid rafts is responsible for the ceramide increase. Elevated ceramide contents stimulate secretory phospholipase A_2_ (sPLA_2_) [340], transcription and translation of which is induced by Stx1 in primary human glomerular epithelial cells together with the cytosolic PLA_2_ (cPLA_2_) [342]. The resulting production of arachidonic acid, in concert with higher cyclooxygenase activity, leads to an increased synthesis of prostacyclin (PGI_2_) and thromboxan A_2_ (TXA_2_) [342]. Free arachidonic acid metabolites activate, e.g., PKC and MAPK and, by modifying gene transcription, cause inflammatory responses. Since PGI_2_ and TXA_2_ partially cause opposite effects, the meaning of these findings for the pathogenesis of Stx-mediated diseases remains unclear [342]. In fact, Stx1-induced TNF-α expression in human monocytes is paralleled by increased prostaglandin E_2_ (PGE_2_) synthesis, but super-induction of PGE_2_ by anisodamin inhibits Stx1-induced TNF-α expression [335].

### 4.7. Interaction of Shiga Toxins with Soluble Factors

In addition to binding to cellular surface receptors, Stxs were also found to directly interact with soluble extracellular protein, i.e., blood constituents. Stx2 activates human complement via the alternative pathway by direct interaction with complement components, considered to be relevant in HUS pathogenesis. Stx2 binds factor H (FH) at short consensus repeats (SCRs) 6–8 and 18–20 and delays and reduces FH cofactor activity on the cell surface [343]. Complement factor H-related protein 1 (FHR-1) and factor H-like protein 1 (FHL-1), similar proteins with regulatory functions, also bind to Stx2. The FHR-1 binding site for Stx2 is located at SCRs 3–5 and the binding capacity of FHR-1*A allotype is higher than that of FHR-1*B. FHR-1 competes with FH for Stx2 binding and thereby reduces FH cofactor activity [344]. Binding to FH depends on the proteolytic cleavage of Stx2A into the A_1_ and A_2_ fragments linked by a disulfide bridge. While binding of FH is exclusively exerted by cleaved Stx2, uncleaved Stx2 binds to human neutrophils and triggers leukocyte/platelet aggregate formation [345]. Furthermore, Stx2 downregulates complement inhibitor CD59 mRNA and protein levels on tubular epithelial and glomerular endothelial cells, and this downregulation is believed to further contribute to complement activation and kidney destruction in EHEC-associated HUS [346]. Even low concentrations of human amyloid P component (HuSAP) inhibit the toxic activity of Stx2 for target cells in vitro and protect mice from lethal effects induced by this toxin [347,348]. The interaction of HuSAP with Stx2 is mediated both by the A subunit and the B pentamer [214]. The soluble extracellular domain of TLR-4 inhibits the binding of Stx2 to neutrophils and the complex Stx2/soluble TLR-4 escapes from capture by HuSAP, allowing the toxin to target and damage human cells [349]. Bovine lactoferrin mitigates the bioactivity of Stx2, but not of Stx1, and this effect at least partly results from degradation of the Stx2 receptor-binding B-subunit [350]. Interactions with these soluble factors, which may occur in the course of STEC-mediated diseases at several stages of the pathogenesis, are likely to interfere with many aspects of Stx–host cell interactions in vitro, although the relevance of this awaits to be fully understood.

### 4.8. Transcellular Transport

The full spectrum of Stx cellular uptake mechanisms also remains to be characterized [8]. Macropinocytosis is an alternative pathway for the uptake of Stxs into cells that do not express Gb_3_ [351]. This pathway in human intestinal epithelial cells, mediated through Src activation, stimulates toxin transcellular transcytosis [352]. Transcytosis across T84 monolayers is facilitated by changes in the actin cytoskeleton induced by latrunculin B, but not cytochalasin D or jasplakinolide. An actin turnover may thus play an important role in Stx1 transcellular transcytosis across intestinal epithelium in vivo since EHEC attachment to epithelial cells causes an actin rearrangement [353]. Neither cell viability nor epithelial barrier function are compromised by this process [28,29]. Differences in translocation rates and directionality, effects of cellular drugs, and competition experiments indicate that Stx1 and Stx2 are transcytosed following different pathways [27,29]. Intracellular toxin is found in association with endosomes, Golgi, ER, and the nuclear membrane, indicative of retrograde transport [30], but the fact that transcytosis is not affected by disruption of the Golgi apparatus [29] implies that transcytosis is independent of retrograde transport [354]. Recent studies indicate that Stx2 can cross enteroid-derived monolayers without loss of epithelial barrier function and still induce stromal cells death [20].

Apparently, toxin uptake by Gb_3_-negative intestinal epithelial cells does not only involve such cells as an inert transport route, but also affects intestinal epithelial cell functions in vitro and in vitro. Stx1 uptake causes galectin-3 depletion from enterocytes by increasing the apical galectin-3 secretion and impairing trafficking of several brush border structural proteins and transporters, including villin, dipeptidyl peptidase IV, and the sodium-proton exchanger 2, a major colonic sodium absorptive protein [352]. The mistargeting of proteins responsible for the absorptive function might be a key event in Stx1-induced diarrhea [352].

## 5. Conclusive Summary

Although primarily recognized as ribotoxic protein synthesis inhibitors, Stxs deploy a plethora of interactions with cells of the STEC-infected host. At first, after released by strictly luminal enteropathogens, the toxins are transcytosed through intestinal epithelial cells partially through Gb_3_-independent epithelial cell damage. Having crossed the intestinal barrier, interaction with Gb_3_/CD77-positive cells results in the generation of host cell-derived microvesicles. Seconded by proteins like soluble TLR-4, serumamyloid P, and complement factor H, binding of Stx to Gb_3_/CD77 on sensitive cells in different organs occurs. Signaling from the cell surface results in internalization and translocation to the cytosol, initiation of endoplasmic and ribotoxic stress responses, and eventually protein synthesis inhibition and intranuclear DNA damage. These effects culminate in cellular death events, mainly involving apoptosis. Furthermore, Stxs can cause alteration of multiple intracellular signaling pathways and thereby disturb intercellular communication implicated in innate immune responses, namely inflammation. Such effects may even extend to alterations of adaptive immune responses. Significant variations in the effects of Stxs on host cells have been observed for different types of cells, tissues, and hosts. Our contemporary understanding of the interactions of Stxs with host cells at the molecular level, reviewed in here, mainly comes from cell cultures studies deploying primary cells and cell lines from human and mice. Despite numerous efforts to establish suitable animal models, none of them completely replicate the EHEC infection and HUS observed in humans patients [12]. However, the pathogenesis of ED, a disease with significant economic impact in pork production and caused by particular STEC variants producing Stx2e, may follow very similar paths as a human EHEC-mediated disease. Intervention measures deduced from an in-depth understanding of this molecular interplay may foster our basic understanding of cellular biology and microbial pathogenesis and pave the way to the creation of host-directed active compounds to mitigate the pathological conditions of STEC infections in the mammalian body.

## Figures and Tables

**Table 1:** Function of amino acid residues in the Shiga toxin B subunits.

Amino Acid in Toxin:			Allocated Function:	Reference:
Stx1	Stx2 ^1^	Stx2e		
(-20)-(-1)	(-19)-(-1)	(-19)-(-1)	signal sequence	[63,73,74]
	Ala1		hydrogen bond to Ser53; part of receptor binding site II	[80]
3–8			β1-sheet	[77]
Cys4	Cys3		disulfide bond to Cys57 (essential for receptor binding); part of receptor binding site II	[76,80,81]
9–14			β2-sheet, interaction with β6-sheet of the adjacent monomer (receptor binding site I ^2^)	[77]
Lys13			part of receptor binding site I ^2^	[82]
10–20			hydrophilic domain: receptor binding, binding of neutralizing antibodies, highly conserved in all Stxs (antigenic domain?)	[76]
Asp16			receptor binding	[83]
Asp17			part of receptor binding site I ^2^	[82,83]
		Asn17	receptor specificity for Gb_4_	[83]
Asp18	Asp17		receptor specificity for Gb_3_/CD77	[81]
20–24			β3-sheet	[77]
Thr21			part of receptor binding site I ^2^	[82,84]
27–31			β4-sheet	[77]
Glu28			part of receptor binding site I ^2^	[82,84]
Phe30			located between receptor binding site I and II; essential for both binding sites	[85]
Asn32			part of receptor binding site II	[84]
Arg33		Arg32	part of receptor binding sites II and III	[80,84,86]
Trp34	Trp33		part of receptor binding site III	[78,80,87]
Asn35	Asn34		part of receptor binding site III?	[80]
36–46			α-helix, forming the central pore of the pentamer together with the α-helices of the other four B subunits; in contact with the anti-parallel α-helix of the A_2_ fragment; conformational changes occur here at low pH (impacting on the translocation of the A_2_ fragment)	[77,79,88]
Ala43		Ala42	receptor binding	[86]
49–53			β5-sheet	[77]
Lys53		Lys52	receptor binding (involved in determination of receptor specificity?)	[81]
	Ser53		hydrogen bond to Ala1; part of receptor binding site II	[80]
Cys57	Cys56		Disulfide bond to Cys4 (essential for receptor binding); part of receptor binding site II	[76,80,81]
Gly60		Gly59	part of receptor binding site I ^2^	[82,86]
65–68			β-sheet, interaction with the β2-sheet of the adjacent monomer (receptor binding site I ^2^)	[77]
Glu65			receptor specificity for Gb3; part of receptor binding site I 2	[82,84]
		Gln64	localization of the toxin in the E. coli cell	[81]
			receptor specificity for Gb4	[83]
		Lys66	receptor specificity for Gb4	[81,83]
Phe68	Phe67	Phe67	receptor binding	[89]
Arg69	Asn68	Asn68	receptor binding	[89]
C-terminal 4 (-5)			receptor binding	[83]

^1^ referring to Stx2 B subunit with 68 amino acids; ^2^ forming hydrogen bonds between polar and acidic side-chains of the B subunits and polar groups of the carbohydrates [77,82].

**Table 2:** Overview of cellular distribution of Gb_3_/CD77-like Stx receptors ^1,2^ in different species.

Cell Type	Man	Mice	Rabbit	Pig	Cattle	Sheep	Goat
**Intestinal epithelial cells**	(+) only for Stx2 [21] + only cell lines [21] + intestinal organoids [20]	+ only distal colon [128]	+ [129,130]	? [131]	+ [132,133]		
Paneth cells	+ [134]						
**Endothelial cells**							
in large blood vessels	+ [118,135,136,137,138]				+ [139]; − [137]		
in the microvasculature	+ [137,140,141,142]						
intestinal	+ [143]		+ [144]		− [131]		
in renal glomeruli	+ [122,145,146]			+ [147]	− [131]		
in the CNS	+ [120,123,124,148]	− [43]	+ [144]	+ [147]			
in the lung		+ [149]					
**Nerve cells**				? [22]			
retinal pigment epithelial	+ [150]						
**Renal tubular epithelial cells**	+ [107,145,151,152,153]	? [154]	− [155]	+ [147]	+ [131,132]		
**Renal glomerular epithelial cells**	+ [156]		− [155]				
**Fibroblasts**							
intestinal myofibroblasts	+ [21]				+ [133]		
**Monocytes/macrophages**	(+) [157] + [158]	+ [159]		+ [147]	+ [160]		
Mesangium cells	(+) [161] + [161,162]		− [155]		− [132]		
Tissue macrophages				+ [147,163]	+ [133]		
**Lymphocytes**				− [147]	+ [164,165]		
B cells	+ [42,166,167]	+ [168]			+ [164,165]		
αβT cells	− [42,166]	− [154,169]			+ [164,165]		
γδT cells					+ [164,165]		
**Granulocytes**	(+) [32]			+ [147]	− [170]	+ [170]	+ [171]
**Erythrocytes**	+ [31,172]			− [22]			
**Hematopoetic stem cells**	+ [173]						
**Platelets**	+ [174,175]						

^1^ Stx2e binding sites / Gb_4_ not considered; for an excellent overview of Stx2e receptor distribution in the tissues of weaned piglets, the reader is referred to Steil et al. [176]; ^2^ Gb_3_ and Gb_4_ are also reportedly synthetized by various human and murine immune cells (reviewed by [177]); however, these findings were not considered if no evidence was found that the molecules function as Stx binding sites in these cells; + = Gb_3_/CD77 and/or Stx binding sites detected; − = Gb_3_/CD77 and/or Stx binding sites not detectable; (+) = evidence for Stx binding sites different from Gb_3_/CD77; ? = indication for the presence of Stx binding sites not yet further biochemically characterized.
